# Roles of the Developmental Regulator *unc-62/*Homothorax in Limiting Longevity in *Caenorhabditis elegans*

**DOI:** 10.1371/journal.pgen.1003325

**Published:** 2013-02-28

**Authors:** Eric L. Van Nostrand, Adolfo Sánchez-Blanco, Beijing Wu, Andy Nguyen, Stuart K. Kim

**Affiliations:** 1 Department of Genetics, Stanford University Medical Center, Stanford, California, United States of America; 2 Department of Developmental Biology, Stanford University Medical Center, Stanford, California, United States of America; University of Michigan, United States of America

## Abstract

The normal aging process is associated with stereotyped changes in gene expression, but the regulators responsible for these age-dependent changes are poorly understood. Using a novel genomics approach, we identified HOX co-factor *unc-62* (Homothorax) as a developmental regulator that binds proximal to age-regulated genes and modulates lifespan. Although *unc-62* is expressed in diverse tissues, its functions in the intestine play a particularly important role in modulating lifespan, as intestine-specific knockdown of *unc-62* by RNAi increases lifespan. An alternatively-spliced, tissue-specific isoform of *unc-62* is expressed exclusively in the intestine and declines with age. Through analysis of the downstream consequences of *unc-62* knockdown, we identify multiple effects linked to aging. First, *unc-62* RNAi decreases the expression of yolk proteins (vitellogenins) that aggregate in the body cavity in old age. Second, *unc-62* RNAi results in a broad increase in expression of intestinal genes that typically decrease expression with age, suggesting that *unc-62* activity balances intestinal resource allocation between yolk protein expression and fertility on the one hand and somatic functions on the other. Finally, in old age, the intestine shows increased expression of several aberrant genes; these UNC-62 targets are expressed predominantly in neuronal cells in developing animals, but surprisingly show increased expression in the intestine of old animals. Intestinal expression of some of these genes during aging is detrimental for longevity; notably, increased expression of insulin *ins-7* limits lifespan by repressing activity of insulin pathway response factor DAF-16/FOXO in aged animals. These results illustrate how *unc-62* regulation of intestinal gene expression is responsible for limiting lifespan during the normal aging process.

## Author Summary

The normal aging process in *Caenorhabditis elegans* involves coordinated changes in expression of about a thousand genes. In order to find upstream regulators responsible for these aging genes, we used a genomics approach to screen for transcription factors that bind to them. We focused on one such regulator, *unc-62/Homothorax*, that encodes a co-factor for a Hox transcription factor. Although essential for development, expression of *unc-62* in adults limits lifespan. We find that *unc-62* is a transcriptional activator of yolk protein genes, which are synthesized in the intestine and encode essential nutrients for progeny but accumulate to toxic levels with age. Additionally, analysis of *unc-62* knockdown indicates that when vitellogenin transcription is decreased, transcription of intestinal genes with somatic functions increases. Thus, activation of yolk protein gene expression by *unc-62* is both a burden on maternal health and a necessary resource for embryonic growth. Surprisingly, we also found that the intestine of old worms has novel expression of non-intestinal proteins that are toxic when expressed in the intestine and are activated by *unc-62* in old age. Our work on transcriptional regulation of intrinsic gene expression differences during normal aging has revealed insights into the mechanisms that limit lifespan.

## Introduction

The normal aging process involves the deterioration of a variety of tissues. In *Caenorhabditis elegans*, this includes loss of mobility and muscle cellular organization, decreased pharyngeal pumping, ectopic neuronal branching, deterioration of synapse function, and degeneration of the intestine [1], [2], [3], [4], [5]. The deterioration of the intestine is particularly striking, as the normal lumenal structure and even entire nuclei are lost in old worms [5]. One approach to understanding the intrinsic process of aging is to find mechanisms responsible for these changes that occur during normal aging [6].

Genome-wide microarray studies of aging in *C. elegans* have identified over a thousand transcripts that significantly increase or decrease in expression with age, covering a wide array of tissue-types and functions [7], [8], [9], [10]. Genes that are altered between young and old include not only downstream effects of aging, but more intriguingly may also reflect changes that are causal for tissue decline. Indeed, variability in the expression of a number of these age-regulated genes predicts remaining lifespan of individual worms, indicating that these genes can serve as biomarkers of physiological aging [11]. However, the genetic and regulatory mechanisms underlying these changes are poorly understood.

In an effort to link gene expression changes during aging to a regulator that mediates longevity, Budovskaya *et al*. identified a hypodermal developmental circuit involving GATA transcription factor genes *elt-3*, *elt-5*, and *elt-6* as an example of a developmental circuit affecting lifespan [10]. Expression of *elt-3* declined with age independently of tested stress and damage signals, and increased activity of *elt-3* led to increased lifespan. These results suggest that mis-regulation of developmental pathways during aging, including the *elt-3* pathway, may be an intrinsic property limiting lifespan of the organism [10]. There are many other transcription factors have been linked to longevity through the insulin signaling, dietary restriction, nutrient sensing, and stress-response pathways that may also be involved in orchestrating the normal aging process (reviewed in [12]).

Here we use a novel genomics approach based upon screening large-scale transcription factor target datasets generated by the modENCODE project to identify potential transcriptional regulators of age-dependent expression in *C. elegans* (E.V.N. and S.K.K. *unpublished data*). The most promising transcription factors identified by this approach include not only regulators involved in stress response pathways, but also developmental regulators. One such factor, UNC-62, is the *C. elegans* ortholog of Hox co-factor Homothorax (Hth/Meis). UNC-62 plays essential roles in development of the nervous system, hypodermis, and vulva [13], [14], [15], [16], but knockdown of *unc-62* beginning in adulthood extends lifespan by ∼40% [17]. Focusing on the intestine, we identified that intestine-specific knockdown of UNC-62 recapitulates this extended lifespan and that UNC-62 decreases expression with age in the intestine. A number of mechanisms can be linked to this extension of lifespan, including shutting off yolk protein (vitellogenin) gene expression, preventing aberrant expression of neuronal genes in the intestine in old age, and increasing transcriptional resources for general intestinal gene expression. These mechanisms illustrate how antagonistic pleiotropy and allocation of cellular resources between somatic health and fertility can play important roles in specifying longevity.

## Results

In order to uncover potential regulators of aging, we identified transcription factors with targets that are enriched for age-regulated genes. To identify targets bound by each transcription factor, we queried the data from 98 ChIP-seq datasets covering 57 transcription factors (many in multiple developmental stages) generated by the *C. elegans* modENCODE consortium [18]. For each ChIP seq experiment, we obtained DNA regions bound by the transcription factor identified by the PeakSeq algorithm (*q*-value<10^−5^) [19]. We noted that for each transcription factor, some binding sites are factor-specific (bound by only a few other transcription factors) whereas others are general (including the extreme case of HOT regions bound by more than >65% of transcription factors assayed)(Figure S1A–S1B) [20]. Compared to general binding sites, factor-specific binding sites are associated with genes whose expression and biological function are more likely to be shared with the transcription factor (E.V.N. and S.K.K. *unpublished data*; [20]). Thus, to improve the association between the presence of a binding site in ChIP-seq data and regulation of expression of nearby transcripts, we narrowed the targets to those that are factor-specific, defined as regions significantly enriched for a given transcription factor and up to 8 other transcription factors out of the 57 total transcription factors assayed by the modENCODE consortium(E.V.N. and S.K.K. *unpublished data*; [20]).

Each set of factor-specific targets was then independently compared to a set of 1106 age-dependent genes obtained from DNA microarray experiments [10], with significant enrichment in the overlap between the two sets identified using Fisher's exact test. At an enrichment cutoff of *p*<10^−5^, we identified 9 transcription factors as potential aging regulators (E.V.N. and S.K.K. *unpublished data*). These include transcription factors that have been previously associated with aging and longevity, such as oxidative damage response factor *skn-1* [21] and developmental regulator *elt-3* [10].

**Figure 1:**
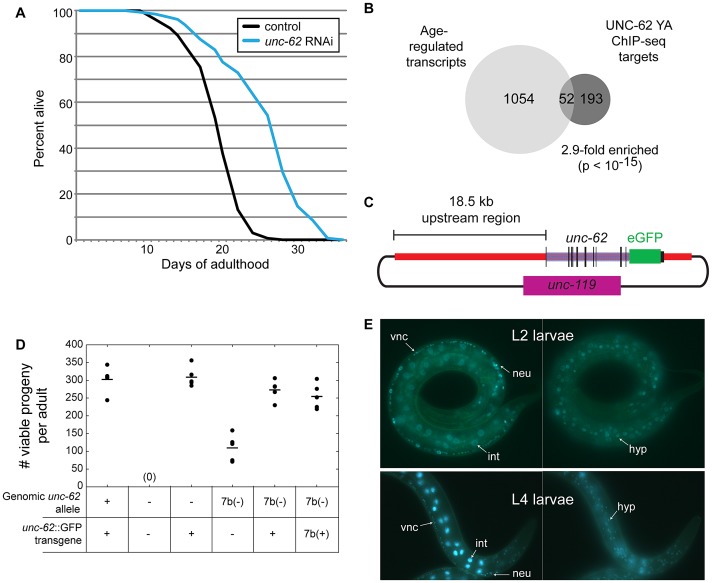
UNC-62 binds age-regulated genes and modulates lifespan. (A) Adult-specific knockdown of *unc-62* extends lifespan by 30%. Approximately 150 day 1 adult wild-type (N2) worms were placed on bacteria expressing dsRNA targeting either *unc-62*, or control bacterial containing an empty vector. The x-axis indicates days of adulthood, and the y-axis indicates the percent of worms that remain alive at that age. The lifespan assay was performed five times (p<10^−5^ for each assay; one representative lifespan assay is shown). See Table S2 for lifespan data. (B) Hox co-factor UNC-62 Homothorax/Meis targets in day 4 of adulthood (Young Adults) show significant overlap with age-regulated transcripts [10], [18]. Only factor-specific binding sites bound by less than 10 out of 57 transcription factors profiled by the modENCODE consortium were utilized. Enrichment *p-*value was determined by Fisher's Exact test. (C) Schematic of the UNC-62:GFP fosmid used to generate an UNC-62 fluorescent reporter. A GFP tag was inserted at the C-terminus of UNC-62 in a fosmid containing all *unc-62* exons and introns, as well as ∼18.5 kb of 5′ promoter sequence. (D) The UNC-62:GFP fosmid-based transgene can rescue the embryonic lethality defects of both an *unc-62* null mutation (*s472;* denoted −) as well as a weaker mutation in *unc-62* exon 7b (*e644*, denoted 7b(−)) [13]. 7b(+) refers to a transgene that expresses only the *unc-62* exon 7b isoform. Circles indicate the number of viable progeny observed from 5 unmated hermaphrodites of each genotype, with the mean indicated by a horizontal line. (E) In hermaphrodites, UNC-62:GFP is expressed in intestine (int), neurons (neu), ventral nerve cord (vnc), vulval precursor cells (not shown), and (right) hypodermis (hyp). UNC-62 intestinal expression is stage-specific: intestine expression in L2 and earlier stages is weak or not visible (top), whereas intestinal expression is dramatically induced by the L4 stage (bottom).

One transcription factor with known roles during embryonic and larval development, *unc-62*, has been shown to modulate lifespan [17] but was not previously linked to changes that occur during the normal aging process. UNC-62 is the *C. elegans* ortholog of *Drosophila* Homothorax and mammalian Meis, which are co-factors for HOX transcriptional regulators [22]. During development, knockdown of *unc-62* activity yields phenotypes in maturation of the vulva, hypodermis, and the nervous system [13], [14], [15], [16]. However, during adulthood, reduction of *unc-62* activity by RNAi extends lifespan by ∼30–40% (Figure 1A) [17]. Using the 1272 UNC-62 binding sites identified by the modENCODE consortium in day 4 young adult worms, we identified 399 factor-specific binding sites associated with 310 target genes. This set of direct targets includes 52 genes that show altered expression with age (2.9-fold enriched, p<10^−15^)(Figure 1B). In this work we characterize a new role for *unc-62* in adults in order to explore the connection between an essential developmental regulator and aging.

### The intestine-specific *unc-62(7a)* isoform decreases with age in the intestine

To investigate the role of *unc-62* in specifying lifespan, we first set out to define the tissues in which it is expressed. Previous experiments using a 2.9 kb proximal promoter for *unc-62* identified expression in vulval precursor cells, neurons, and some hypodermal cells [15]. Additionally, SAGE-tag sequencing from dissected intestines showed that *unc-62* is expressed in the intestine [23]. In order to characterize the expression of *unc-62* further, we made use of a GFP reporter for *unc-62* in its full genomic context. This strain was generated by creating a modified fosmid with GFP inserted at the C-terminus of UNC-62, and includes regulatory elements contained within introns as well as distal promoter elements (Figure 1C). Biolistic bombardment was then used to obtain a strain expressing a low-copy, integrated UNC-62:GFP transgene. This fusion was sufficient to rescue embryonic and larval lethality phenotypes of the *unc-62* deletion allele (*s472*)(Figure 1D).

Using this translational fusion, we observed strong UNC-62:GFP expression in a variety of tissues in the hermaphrodite, including the vulval precursor cells, the ventral cord motorneurons and other neurons, the hypodermis, and the intestine (Figure 1E). Expression in neurons and hypodermis is visible throughout development and into adulthood; expression in vulval precursor cells is only seen when that lineage emerges in the L3 and L4 stages (data not shown). For the intestine, we observed strong expression beginning in the L3 stage and continuing into adulthood (Figure 1E).

**Figure 2:**
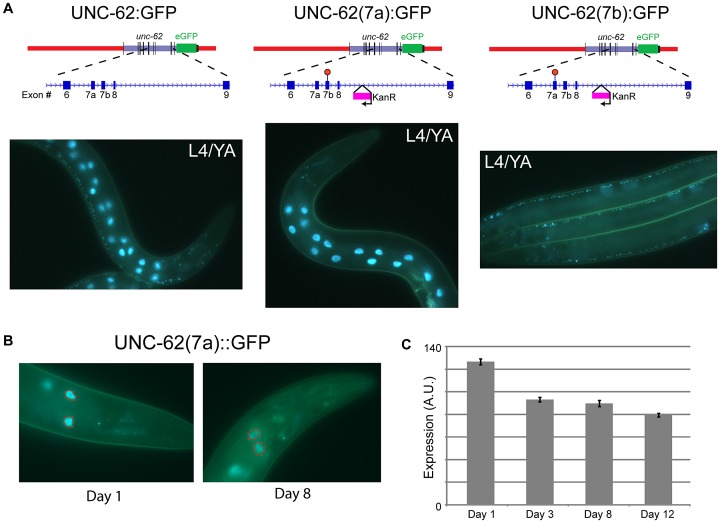
Alternative splicing of UNC-62 generates intestine-specific *unc-62(7a)* and neuronal/hypodermal-specific *unc-62(7b)* isoforms. (A) Strains expressing isoform-specific reporters for *unc-62(7a)* and *unc-62(7b)* show stage- and tissue-specific expression. (top) Beginning with the transgenic fosmid described in Figure 1C, stop codons were inserted into *unc-62* exons 7a or 7b by site-directed mutagenesis to obtain isoform-specific reporters. During this process, a kanamycin resistance cassette was inserted into the eighth intron of *unc-62*. (bottom) Alternative isoforms of UNC-62 show tissue-specific expression in adults. (left) UNC-62:GFP is observed in intestinal, neuronal, and hypodermal cells. (center) UNC-62(7a):GFP is highly expressed in the intestine in the L4 larval stage and young adults, but is not visible in other tissues. (right) UNC-62(7b):GFP is not observed in the intestine, but is expressed in the hypodermis (not shown), the ventral nerve cord, and other neurons. Strains were imaged in a *glo-4(ok623)* background to limit gut autofluorescence. (B) UNC-62(7a):GFP expression decreases between day 1 and day 8 of adulthood. Strain contains *glo-4(ok623)* to reduce gut autofluorescence, and expression was quantified only in the first pair of intestinal nuclei (dotted red circles). (C) Quantification of UNC-62(7a):GFP as shown in (B). Bars indicate mean fluorescence (in arbitrary units) observed from populations of at least 32 worms measured at different days of adulthood, with error bars indicating standard error of the mean. Day 1 expression was significantly higher compared to expression in days 3, 8, or 12 (*p*<10^−4^ by Student's t-test) in two independent experiments (see Figure S2).

Alternative RNA splicing of *unc-62* produces isoforms that differ in usage of the seventh exon (7a and 7b), which encodes the N-terminal region of the UNC-62 TALE homeodomain [13]. Transcripts including exon 7a increase in abundance more than 100-fold between embryos and adults, whereas transcripts containing 7b are expressed roughly equivalently throughout development [13]. To query the tissue-specific expression of these isoforms, we generated UNC-62:GFP reporters that express only UNC-62(7a) or UNC-62(7b). These reporters were made by inserting a stop codon in exon 7b into the UNC-62:GFP translational reporter such that it can only express UNC-62(7a):GFP, or a stop codon in exon 7a such that the reporter can only express UNC-62(7b):GFP (Figure 2A). Strains stably expressing either of these isoform-specific GFP reporters were then generated by biolistic bombardment. Fluorescent imaging of these strains indicated that UNC-62(7a) was predominantly expressed in the intestine starting in L3 and continuing through adulthood. In contrast, UNC-62(7b) was expressed in neurons, the ventral nerve cord, vulval precursor cells, and hypodermis beginning in embryos and continuing through adulthood (Figure 2A).

We next determined whether expression of the *unc-62* isoforms change with age in the intestine. We measured expression of the intestinal *unc-62(7a)* isoform during normal aging, and observed that it decreases in the intestine by day 8 of adulthood (∼26% decrease, p<10^−10^)(Figure 2B–2C, Figure S2A). A similar decrease was observed in whole-worm *unc-62(7a)* mRNA levels between day 2 and day 8 of adulthood when measured by qPCR (Figure S2B). In contrast, *unc-62(7b)* was not observed in the intestine at any age. Thus, the decrease in expression of intestinal *unc-62(7a)* with age is not caused by changes in alternative splicing, but rather by a decrease in the level of transcription.

### *unc-62* acts in the intestine and hypodermis to modulate lifespan

**Figure 3:**
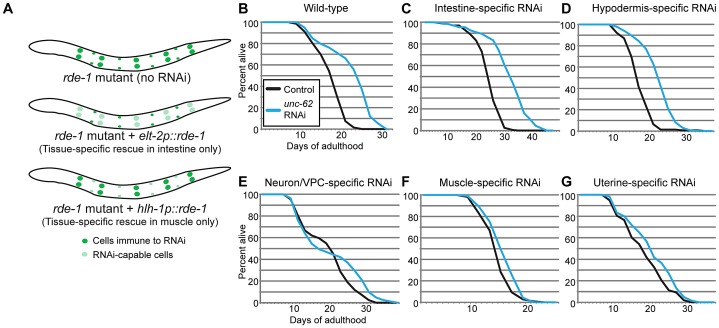
UNC-62 knockdown extends lifespan in intestinal and hypodermal tissues. (A) Tissue-specific RNAi is achieved in worms by starting with a strain deficient for the RNAi pathway due to the *rde-1(ne219)* mutation. A transgene expressing *rde-1* under a tissue-specific promoter (examples shown use *elt-2* to drive intestine-specific expression, or *hlh-1* to drive muscle-specific expression) rescues the RNAi pathway only in the desired tissue. (B) *unc-62* RNAi extends lifespan by ∼30% (*p<*10^−5^) in wild-type worms. (C–D) A ∼30% extension is observed when *unc-62* is knocked down in (C) a strain expressing *rde-1* from the *elt-2* intestinal promoter (strain OLB11) or (D) the *lin-26* hypodermal promoter (strain NR222). (E–G) In contrast, *unc-62* knockdown in (E) neurons and vulval precursor cells (a short *unc-62* promoter in an *rde-1(ne219);rrf-3(pk1426)* background; strain NK742), (F) muscle (*hlh-1* promoter; strain NR350), or (G) uterine cells (*fos-1A* promoter in an *rde-1(ne219);rrf-3(pk1426)* background; strain NK640) did not significantly extend lifespan (*p>*0.01). The *rrf-3(pk1426)* mutation provides increased RNAi sensitivity in neuronal cells. Except for uterine-specific RNAi, lifespans were performed two or more times (Table S2). Lifespan data shown is aggregated from multiple simultaneous experiments.

As *unc-62* is expressed in a variety of tissues, we wanted to identify the tissues in which *unc-62* functions to affect lifespan of the entire organism. To do this, we performed RNAi knockdown of *unc-62* in strains that generate an RNAi response only in specific tissues. These strains contain a mutation in the RNAi pathway (*rde-1*) that leads to inactivation of the RNAi response, as well as a transgene expressing *rde-1* under a tissue-specific promoter to restore the RNAi response only in that tissue (Figure 3A) [24], [25]. We found that knockdown of *unc-62* either only in intestine or only in the hypodermis was sufficient to cause a ∼30% extension of median lifespan (Figure 3C–3D). As a control, we crossed in the *unc-62*:GFP transgene and observed that GFP RNAi reduced GFP expression only in the proper tissue for the intestinal- and the hypodermal-specific RNAi strains (Figure S3). However, *unc-62* RNAi did not have a significant effect on longevity in muscle tissue, neuronal tissue or the uterus (Figure 3E–3G). These results indicate that wild-type *unc-62* activity in the intestine and hypodermis is critical for its role in limiting wild-type lifespan. In this work, we primarily focus on intestinal activity of *unc-62* and the intestine-specific *unc-62(7a)* isoform.

Next, we asked at what time *unc-62* acts to limit lifespan. Loss of *unc-62* during development leads to severe developmental defects, indicating that *unc-62* is beneficial up until adulthood. In contrast, *unc-62* RNAi started at the first day of adulthood extends lifespan (Figure 1A). We found that *unc-62* RNAi beginning at day five of adulthood could still significantly extend lifespan after the end of self-fertility (Figure S4A). This indicates that even after day five, *unc-62* still performs functions that are detrimental and that limit the lifespan of wild-type worms.

### Stage-specific binding of targets by the UNC-62 Homothorax transcription factor

**Figure 4:**
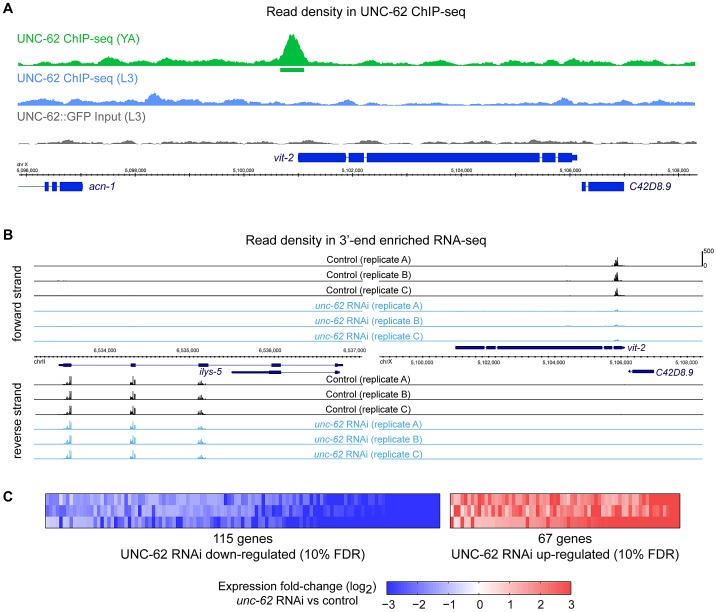
Identification of direct and regulated targets of UNC-62 in adults by ChIP–seq and RNA–seq. (A) An example of UNC-62 ChIP-seq read density at a significantly enriched binding site is shown. Top tracks show read density in ChIP-seq experiments for UNC-62 in young adults (green) and L3 larvae (blue) as well as non-immunoprecipitated input control (grey). Boxes underneath the read density tracks indicate significant binding sites (*q*-value≤10^−5^). Bottom tracks indicate genes (with coding exons in thick blue boxes). (B) Examples of *unc-62* RNAi 3′-end enriched RNA-seq data is shown. We performed three independent experiments in which we fed worms either *unc-62* RNAi or control bacteria, isolated mRNA and generated RNA-seq libraries, and sequenced these libraries on the Illumina HiSeq platform. For the *ilys-5* (left) and *vit-2* (right) genomic regions, reads map to annotated exon regions on the proper strand, and are enriched at the 3′ end of the transcript. Read densities are displayed for control (black) and *unc-62* RNAi (blue), scaled as reads per million uniquely mapping reads. (C) Rank Products-based analysis (based on [27]; see Methods and Figure S5) to identify genes reproducibly altered across all three biological replicates identified 67 genes significantly increased and 115 genes significantly decreased upon *unc-62* RNAi at a 10% false positive rate.

In addition to the adult ChIP-seq targets described earlier, we analyzed UNC-62 ChIP-seq targets identified in the earlier L3 larval stage, when *unc-62* appears predominantly as the 7b isoform (E.V.N. and S.K.K. *unpublished data*). In the L3 stage, the ChIP seq experiments identified 1193 UNC-62 binding sites, of which 251 are factor-specific. However, many young adult UNC-62 binding sites showed little enrichment in the L3 stage ChIP-seq experiment (Figure 4A and E.V.N. and S.K.K. *unpublished data*). We compared the 151 genes associated with these UNC-62 L3 binding sites to the set of aging-regulated genes and found only 10 UNC-62 L3 targets that are also age-dependent, which is not significantly enriched (.98-fold, p>.1). Thus, although the adult UNC-62 targets tend to be differentially expressed with age, the UNC-62 targets in L3 larvae are not. The genes bound by UNC-62 also have differential tissue-specificity: L3 larval targets are enriched for neuronal-enriched expression, whereas young adult targets are instead enriched for intestine-specific expression (E.V.N. and S.K.K. *unpublished data*). These distinctions corresponds to the pattern of alternative splicing of UNC-62; i.e., up until the L3 stage *unc-62* is expressed predominantly as the *unc-62(7b)* isoform in neurons and the hypodermis, whereas in adults *unc-62* appears as the *unc-62(7a)* isoform in the intestine and as the *unc-62(7b)* isoform elsewhere (E.V.N. and S.K.K. *unpublished data*). We focused our analysis on the young adult targets as these targets are enriched for genes with age-dependent expression.

### UNC-62 is a direct and necessary activator of intestinal yolk protein genes

To understand the molecular effects of *unc-62* knockdown in adults, we identified the targets of UNC-62 that are altered when *unc-62* expression is knocked down. Poly(A)+ RNA was prepared from replicates of young adult hermaphrodites grown on either *unc-62* RNAi or empty vector RNAi. We then prepared 3′ end-enriched RNA-seq libraries [26], with each sample barcoded to allow for multiplex sequencing. The triplicate libraries of empty vector and *unc-62* RNAi were separately pooled and sequenced, yielding >14 million mapped reads for each sample (Figure 4B and Table S1).

To identify transcripts with consistently altered expression upon *unc-62* RNAi treatment, we developed an approach based upon a Rank Product method used for DNA microarray analysis (Figure S5) [27]. Our analysis yielded 182 transcripts with altered expression upon *unc-62* RNAi treatment at a false discovery rate of 10%, with 115 transcripts showing decreased expression and 67 showing increased expression upon *unc-62* RNAi (Figure 4C and Dataset S1). Of these 182 *unc-62* dependent transcripts, only ten are directly bound by UNC-62 in young adults (factor-specific binding sites with *q*-value<10^−5^) indicating that the remainder are indirect targets (Table S3).

**Figure 5:**
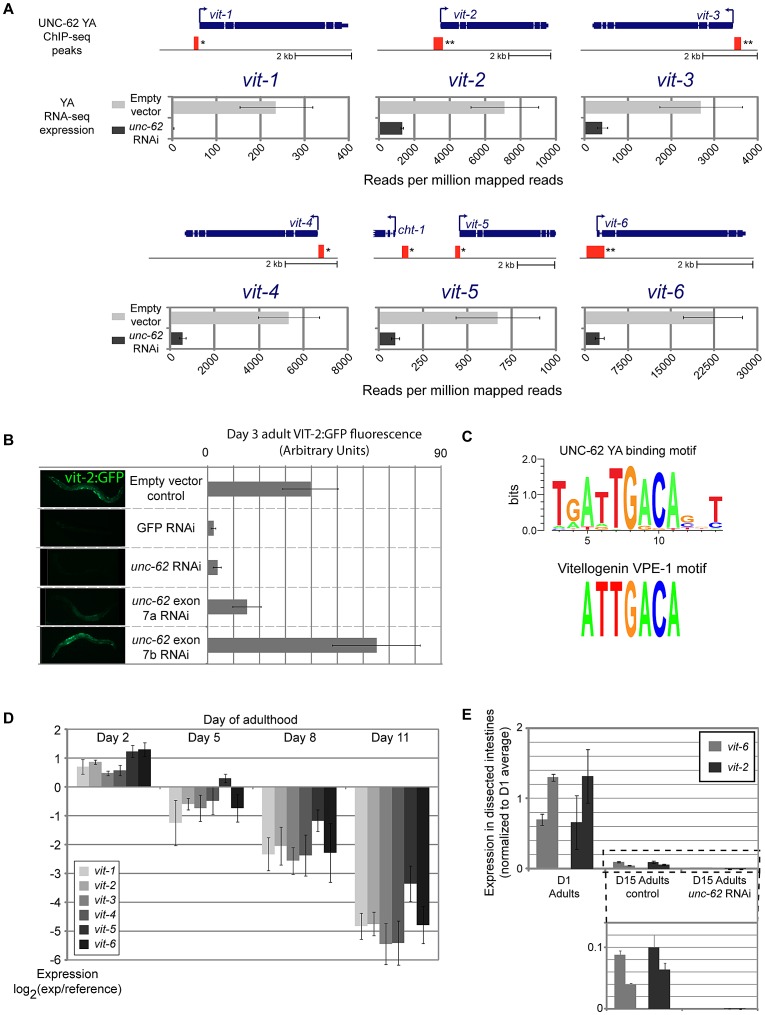
UNC-62 binds to and activates expression of all six *C. elegans* yolk protein genes. (A) The thick and thin blue lines indicate the exon and intron structure of each of the six vitellogenin loci. Red boxes indicate the position of regions significantly enriched in UNC-62 ChIP-seq of day 4 adult (YA) worms. (*) indicates *q*-value<0.001 and (**) indicates *q<*10^−5^ binding sites. Below each gene, RNA-seq results are displayed for worms fed either *unc-62* RNAi or control bacteria. Bars indicate the mean, and error bars the standard deviation, of sequencing read density (reads per million mapped reads) for vitellogenin genes in triplicate RNA-seq experiments. (B) Bars indicate mean, and error bars indicate standard deviation, of measured fluorescence of a *vit-2:GFP* reporter under various RNAi conditions. Expression in ∼15 worms was quantified using ImageJ. (C) (top) A *de novo* motif search in UNC-62 young adult binding sites with RSAT [58] identifies a TGATTGACA motif as the prominent sequence motif. (bottom) This motif is similar to the ATTGACA VPE-1 vitellogenin regulatory motif previously described [30]. (D) All six vitellogenin genes decrease expression with age (as assayed by whole-worm microarrays of 2, 5, 8, and 11 day old adult worms [10]). Bars indicate mean expression observed across replicated arrays, with error bars indicating standard deviation. (E) UNC-62 activates vitellogenin expression in old adults. Bars indicate expression of two biological replicates of *vit-2* (black) and *vit-6* (grey), determined by qRT-PCR of RNA isolated from ∼100 dissected intestines of day 1 adults and day 15 adults fed either control or *unc-62* RNAi bacteria. Error bars indicate standard deviation of triplicate qPCR technical replicates. Statistical significance is not indicated as this experiment included only two biological replicates.

We found that three of the eight genes that are bound and activated by UNC-62 are vitellogenin genes. *C. elegans* contains 6 vitellogenin transcripts (*vit-1* through *vit-6*) that encode the major yolk proteins [28]. These transcripts are specifically expressed in the intestine during the reproductive period, and vitellogenin proteins are exported out of the intestine into oocytes [29]. RNA-seq experiments indicated that expression of all six vitellogenin transcripts is strongly dependent on *unc-62* activity, as there is a 4–10-fold decline in expression for *vit-2*, *vit-3*, *vit-4*, *vit-5* and *vit-6* and a ∼100-fold decline for *vit-1* in *unc-62* RNAi worms as compared to controls (Figure 5A). *unc-62* RNAi also results in a strong decrease in the level of expression of a VIT-2:GFP translational reporter, indicating that protein as well as mRNA levels of *vit-2* are dependent upon UNC-62 activity (Figure 5B). RNAi targeting exon 7a of *unc-62* results in a decrease of VIT-2:GFP expression, whereas RNAi targeting exon 7b does not, indicating that the *unc-62(7a)* isoform activates vitellogenin expression (Figure 5B).

In addition to the significant association observed at three of the six vitellogenin loci in the initial ChIP-seq analysis (*q*-value<10^−5^)(Table S3), we found that the remaining three vitellogenin loci were bound by UNC-62 in adults with *q-value*<0.01 (Figure 5A). Interestingly, interaction with vitellogenin promoters is not observed in ChIP-seq of UNC-62 in L3 stage worms, indicating that UNC-62 is bound to the vitellogenin promoters in adults but not young larvae. We validated binding of UNC-62 to the promoter region of the *vit-2* and *vit-*6 genes in adults by ChIP-qPCR, observing 5- to 15-fold enrichment of UNC-62 binding to these regions compared to control promoter regions (Figure S6).

Previous experiments have identified two sequence motifs that are critical sequence elements shared among all 6 *vitellogenin* promoters: GATA-motif VPE-2 (CTGATAA) and VPE-1 (ATTGACA) [30]. The VPE-1 motif contains the core TGACA sequence bound by Homothorax in *Drosophila* [31]. We used a *de novo* motif search among all UNC-62 young adult binding sites and found that a motif with consensus sequence ATTGACA was the most significantly enriched motif, and that the seven base ATTGACA sequence itself was 4.3-fold enriched in UNC-62 binding sites (Figure 5C, Figure S7). The finding that UNC-62 is a direct and necessary regulator of all six vitellogenin genes, combined with the fact that the VPE-1 motif perfectly matches the UNC-62 motif identified from a *de novo* motif search, suggests that UNC-62 directly binds to the VPE-1 element and activates vitellogenin expression.

The six vitellogenin genes are among the genes that show the greatest decrease in expression with age in the entire *C. elegans* genome, with a 16-fold decrease in expression between young (day 2) and old (day 11) adulthood (Figure 5D) [10], [32]. Having observed that UNC-62 binds to and activates the vitellogenin genes in young adults, we next asked whether residual levels of vitellogenin expression in old adults were still dependent on UNC-62 activity. We micro-dissected the intestine from day 15 adult worms fed either control or *unc-62* RNAi bacteria, and performed qRT-PCR on RNA isolated from the intestines to measure levels of *vit-2* and *vit-6* expression. Despite expression levels decreasing with age, *vit-2* and *vit-6* expression declined further when *unc-62* was knocked down in old adults (Figure 5E), consistent with residual vitellogenin expression in old age remaining activated by *unc-62*.

### Indirect targets of *unc-62* are enriched for genes altered during aging

**Figure 6:**
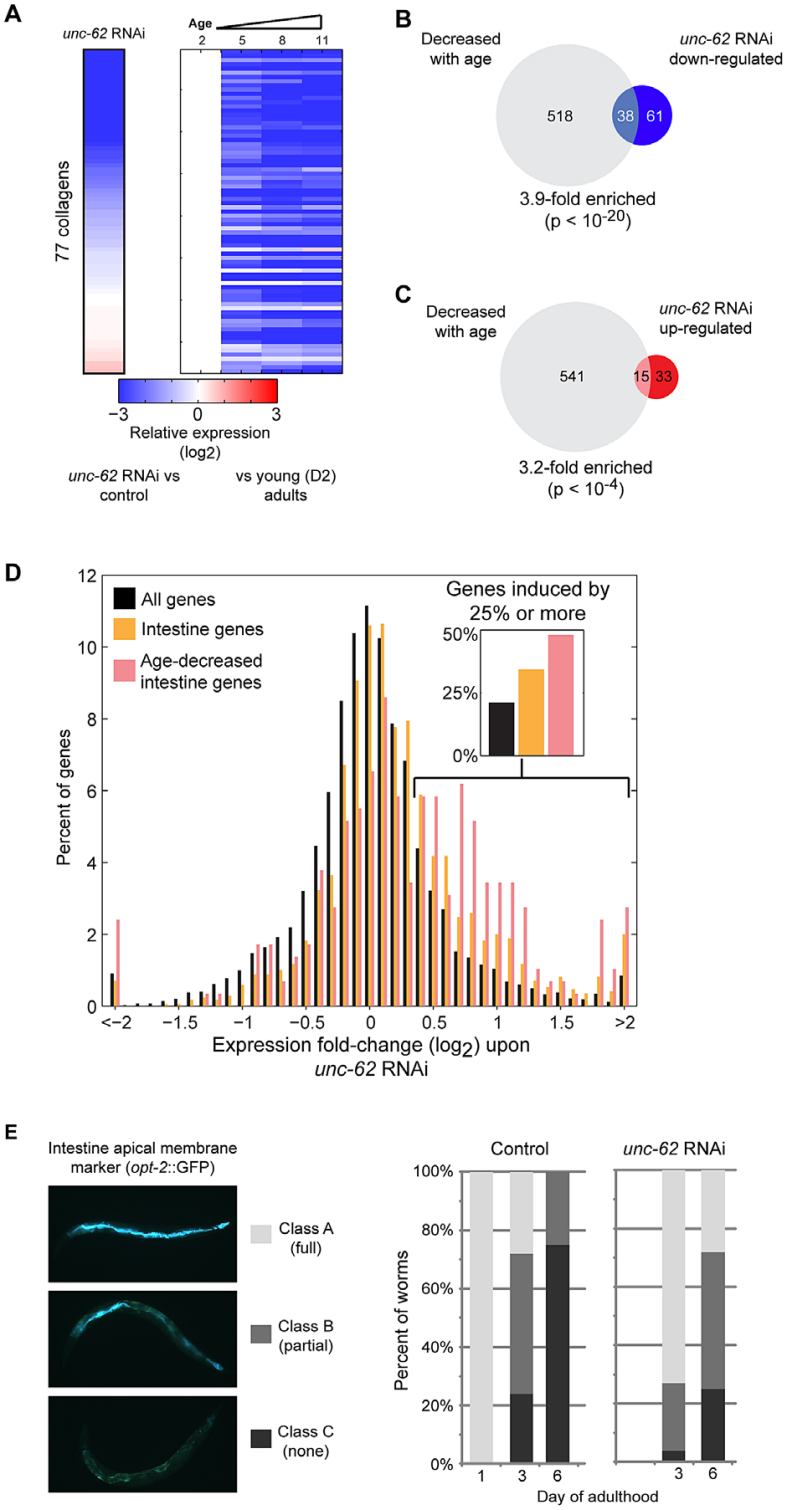
UNC-62 RNAi decreases expression of collagen genes and increases expression of intestinal genes. (A) *unc-62* RNAi decreases expression of collagen genes. Rows correspond to 77 collagen genes profiled in both our RNA-seq experiment as well as microarray studies of aging. Each row shows data for an individual collagen gene. (left) Color indicates RNA-seq fold-change between *unc-62* RNAi and control, averaged from triplicate biological experiments. (right) The four columns indicate expression at day 2, 5, 8, and 11 from whole-worm microarrays (normalized to day 2 expression) (data from [10]). (B) Of the 99 genes down-regulated upon *unc-62* RNAi (at 10% FDR) profiled by aging microarrays, 38 also decrease with age (3.9-fold enriched, p<10^−20^ by Fisher's exact test). (C) The 48 genes that increase in expression upon *unc-62* RNAi (10% FDR) show a significant overlap with the 556 genes that decrease in expression with age (p<10^−4^) [10]. (D) *unc-62* RNAi causes a broad increase in expression of intestinal genes. The histogram indicates the distribution of genes by average fold-change upon *unc-62* RNAi. For 1699 genes with intestine-enriched expression [23], [33], [34] as well as the subset of 291 genes that also decrease expression with age [10], we determined the average mean-centered fold-change from triplicate RNA-seq experiments of *unc-62* RNAi as compared to controls. Both intestine-enriched genes (orange) and intestine-enriched genes that decrease expression with age (pink) are significantly shifted towards increased expression upon *unc-62* RNAi (*p-*value<10^−20^ by Kolmogorov-Smirnov test). (insert) The percent of genes with fold-change of 1.25 or more are indicated. (E) (left) An OPT-2:GFP reporter was used to measure intestinal morphology. In young adults, *opt-2* localizes to the apical membrane of the intestine (class A). This structure deteriorates in older worms, as some worms contain the structure through only a portion of their body (class B) and others lack *opt-2* expression altogether (class C). (right) Worms were placed on either *unc-62* or control RNAi at day 1 of adulthood, and ∼30 worms for each were imaged and annotated at day 3 and day 6. Stacked bars indicate the percent of worms that were observed as class A (light grey), class B (dark grey), or class C (black).

To further understand the roles of *unc-62* during aging, we analyzed the 182 transcripts that show altered expression in *unc-62* RNAi-treated worms, consisting of 67 transcripts that increase expression and 115 transcripts that decrease expression. The 115 targets that are (directly or indirectly) activated by wild-type *unc-62* show a strong enrichment for genes specifically expressed in the hypodermis (2.6-fold; p<10^−5^). Notably, 41 of these 115 genes are collagen genes, out of a total of 90 collagen genes quantified in the RNA-seq experiment (29-fold enriched, *p*<10^−15^)(Figure 6A). These *unc-62-*activated genes also include 38 that decrease in expression during normal aging (a 3.9-fold enrichment, *p*<10^−20^) [10], including 26 of the 41 *unc-62*-activated collagen genes (Figure 6A–6B). These findings suggest that changes in gene expression of hypodermal genes upon *unc-62* knockdown (particularly, collagen genes) are similar to changes normally observed as adult worms age; i.e., *unc-62* RNAi mimics the normal aging process in the hypodermis.

Next, we considered the genes with increased expression upon *unc-62* RNAi. At the tissue-level, we found that 32 of these genes show intestine-enriched expression in one or more datasets (2.6-fold enriched, p<10^−10^). These genes that are repressed by wild-type *unc-62* activity (although not bound by UNC-62 directly) also show a 3.2-fold enrichment for genes that decrease in expression with age (p<10^−4^)(Figure 6C). Thus, *unc-62* RNAi indirectly leads to increased expression of genes that normally decline with age and are primarily expressed in the intestine.

A possible explanation for the overall increase in gene expression in the intestine in *unc-62* RNAi worms could be that it is an indirect consequence of shutting down vitellogenin gene expression. The vitellogenin transcripts are among the most highly-expressed transcripts in *C. elegans*; *vit-4* and *vit-6* are the sole non-ribosomal protein genes among the ten highest expressed genes in young adults, and the six vitellogenin genes comprise ∼3% of all mapped reads in young adults in RNA seq experiments despite being expressed solely in intestinal cells (Table S1). Thus, as an indirect consequence of dramatically reducing vitellogenin gene expression by *unc-62* RNAi, transcriptional machinery that was previously allocated to express vitellogenin genes may become available to activate transcription of other genes.

This model posits that the decrease in vitellogenin transcription would lead to a generalized increase of the remaining genes expressed in the intestine. To test this, we identified 1699 genes that are intestine-enriched [23], [33], [34] as well as the subset of 291 that decrease in expression with age [10]. We observed that intestinal genes in general, and particularly those that decrease expression with age, are significantly shifted towards increased expression upon *unc-62* RNAi (both *p*<10^−20^ by Kolmogorov-Smirnov test)(Figure 6D). In contrast, there was no shift observed either for neuron-enriched or muscle-enriched genes (*p*>0.01 by Kolmogorov-Smirnov test)(Figure S8). Thus, in addition to the 67 genes that were identified to be significantly altered upon *unc-62* RNAi, there is a general increase in expression of intestinal genes in *unc-62* RNAi worms that may provide an indirect benefit contributing to longer life. As intestinal gene expression generally declines with age, this broad effect of gene activation by *unc-62* RNAi opposes the normal aging process in the intestine.

The above findings suggest that *unc-62* knockdown may delay the age-related decay of the intestine. In order to assay the morphology of the intestine, we examined intestinal expression of an *opt-2*:GFP reporter. *opt-2* encodes an intestinal low affinity/high capacity oligopeptide transporter that localizes to the apical membrane of the intestine, and is visible as a single tubular structure tracking with the inner intestinal membrane in young adults (Figure 6E) [35]. During normal aging, this pattern rapidly degrades by day three of age (long before worms begin to die from old age). In *unc-62* RNAi worms, the age-related deterioration of intestinal morphology (measured by presence of apical expression of *opt-2:GFP*) is delayed (Figure 6E). In summary, knockdown of *unc-62* increases lifespan, increases the period of healthy intestine morphology, and ameliorates intestinal expression changes that occur during wild-type aging.

### Expression of a new class of UNC-62 targets in the intestine in old age

As part of our analysis, we discovered a set of genes bound by UNC-62 in young adults that, during development, are enriched for expression in neuronal cells as opposed to the intestine. We next examined whether expression in the intestine of these targets changes during normal aging, and whether their expression is dependent on *unc-62* activity.

To profile expression changes with age in the intestine, we micro-dissected young and old intestines, isolated RNA, and performed qPCR to assay expression of these UNC-62 targets. In these samples, control transcripts known to be specifically expressed in the intestine showed a greater than 10-fold enrichment compared to non-intestinal transcripts (Figure S9). We randomly selected eight UNC-62 young adult targets (that we refer to as Class N) that have neuronal-enriched expression patterns but had significant UNC-62 binding in adults by ChIP-seq, allowing for the possibility that their expression is under UNC-62 control during normal adult aging. As negative controls, we used ten genes with neuronal-enriched expression that were bound by UNC-62 in early development, but no longer identified as significantly bound in adults.

**Figure 7:**
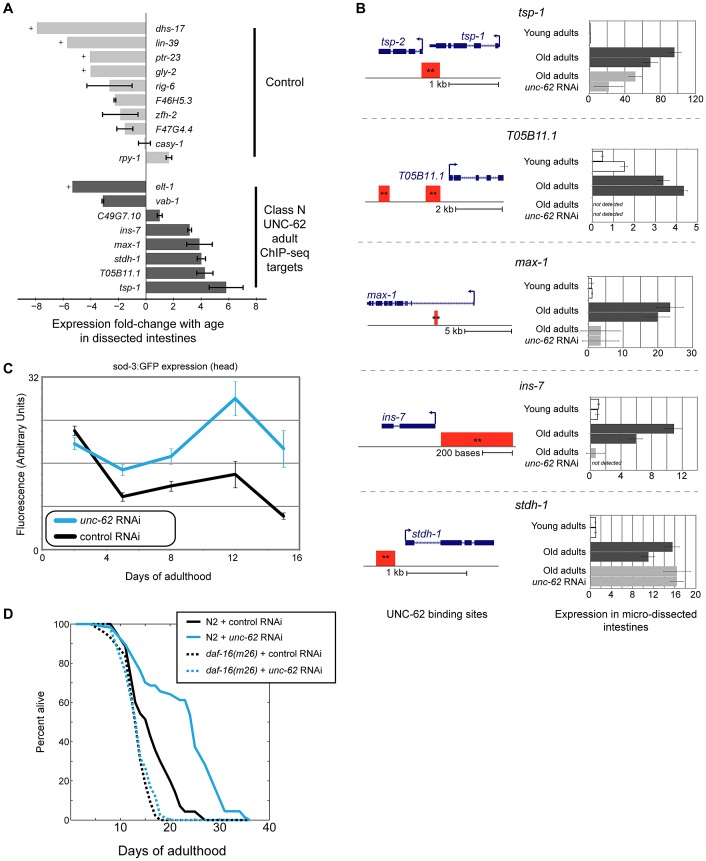
*ins-7* and other *unc-62-*dependent class N targets are activated in the old intestine. (A) Class N *unc-62* targets tend to increase with age in the intestine. Eight class N targets (bound by UNC-62 in the young adult with neuronal-enriched expression in development (dark grey bars)), as well as ten control targets that have neuronal-enriched expression and are bound by UNC-62 in L3 stage but not the adult stages (light grey bars), were assayed for age-related changes in the intestine (between day 1 and day 15 adults). Expression in dissected intestines was quantified by qPCR as described in Methods using a beta-tubulin control (C36E8.5). Bars indicate the ratio of the expression between young and old as measured by the ΔΔCt method, and error bars indicate standard deviation of technical triplicate qPCR measurements. + indicates transcripts that were quantified in young but not detected in old intestines; for these transcripts, age-downregulation was calculated using a Ct value of 40 as an upper bound of old intestinal expression. (B) *unc-62* activates four of five class N targets in old intestines. For the five UNC-62 adult targets that increased with age by more than two-fold, we analyzed expression in day 1, day 15 control, and day 15 *unc-62* RNAi micro-dissected intestines (two biological replicate samples each). (Left) Schematics for these five genes indicates gene structures (in blue), as well as associated UNC-62 binding sites in young adult (red) ChIP-seq. All binding sites shown were factor-specific and significant at *q*-value 10^−5^ (**). (Right) Expression fold-change was calculated using a beta-tubulin control as before, and are shown relative to the average expression between young intestine replicates. For four of the five (*tsp-1*, *max-1*, *ins-7*, and T05B11.1), expression in old intestines was diminished when *unc-62* was knocked down. *unc-62* RNAi had no effect on *stdh-1* expression in old intestines. Although the binding site overlapping the 3′ end of *tsp-1* was associated with *tsp-1* using our analysis pipeline, we found that *tsp-2* showed a similar expression pattern in dissected intestines (Figure S10). (C) *unc-62* RNAi activates activity of main insulin pathway target DAF-16/FOXO. We quantified expression of *sod-3:GFP*, which is a reporter of DAF-16 activity [40]. Approximately 25 worms were imaged for each aging timepoint (x-axis), and fluorescence in the head (y-axis) was quantified using ImageJ. Error bars indicate standard error of the mean. (D) *unc-62* RNAi extends lifespan in wild-type worms (37% increase in mean lifespan, *p*<10^−5^), but not in *daf-16(m26)* mutants (3% increase, *p*>0.1). X-axis indicates days of adulthood, and the y-axis indicates percent of worms remaining alive. Similar results were observed in a replicate experiment using *daf-16* RNAi (lifespan data in Table S2).

We observed that expression of the eight UNC-62 Class N young adult targets in dissected intestines showed a significant shift towards increased expression in old age (p = 0.013 by Wilcoxon rank-sum test)(Figure 7A). Specifically, five of the eight adult targets increased more than two-fold in expression with age (including one gene (*ins-7*) which has previous been shown to increase with age in the intestine [36]), whereas none of the ten control targets did so. To determine whether expression of class N genes was dependent on UNC-62 activity, we obtained RNA from micro-dissected intestines from old adults grown on *unc-62* RNAi. We found that four (*tsp-1*, *max-1*, *ins-7*, and *T05B11.1*) of the five class N genes that increase expression by two-fold in intestines with age showed decreased expression upon *unc-62* RNAi (Figure 7B). In summary, class N genes are an intriguing set of *unc-62*-dependent genes that are expressed predominantly in neuronal tissues in development, but then become expressed in the intestine in old adults.

Late-life expression of at least two class N genes (*max-1* and *ins-7*) appears to be detrimental, as RNAi treatment or loss-of-function mutations in these two genes have been shown to extend lifespan. The first, *max-1*, encodes a PH/MyTH4/FERM domain-containing protein that is required for proper motor axon projections [37], and *max-1* RNAi treatment increases lifespan by ∼5% [38]. The second, *ins-7*, encodes an insulin/IGF-1-like peptide that acts as an agonist for the insulin/IGF-1 receptor DAF-2 [39]. The insulin/IGF-1 signaling pathway ultimately represses the activity of the FOXO-family transcription factor DAF-16. When the activity of the insulin/IGF-1 signaling pathway is low or off, DAF-16/FOXO localizes to the nucleus and activates expression of beneficial genes that extend lifespan [12]. Knockdown of insulin *ins-7* activity throughout the worm results in activation of DAF-16/FOXO and extends lifespan, and intestine-specific over-expression of *ins-7* in young adults is toxic [36]. We verified that knockdown of *ins-7* expression specifically in the intestine can extend lifespan by showing that intestine-specific RNAi of ins-7 results in an 8.3% extension of mean lifespan (*p = *7.8×10^−8^ by log-rank test)(Figure S4B).

One possible mechanism contributing to the lifespan extension seen in *unc-62* RNAi worms is that *ins-7* expression does not increase, but instead remains at a low level in the intestine in old age. As a result, DAF-16/FOXO activity would not be repressed by *ins-*7, and could instead remain high in *unc-62* RNAi worms as opposed to being repressed in normal aged worms. According to this possibility, *unc-62* RNAi should result in activation of DAF-16/FOXO activity and the longevity phenotype should be at least partially dependent on *daf-16* activity. First, we found that *unc-62* RNAi results in increased expression of a DAF-16 reporter (*sod-3:GFP* [40]) in aged animals, suggesting an increase of DAF-16/FOXO activity (Figure 7C). As has been previously observed, increased DAF-16/FOXO activity upon *unc-62* knockdown was not observed in young adults (when *ins-7* is not yet induced in the intestine) [17]. Second, we found that a *daf-16* mutation suppresses the longevity caused by *unc-62* RNAi, indicating that the benefit conferred by *unc-62* knockdown requires *daf-16* activity (Figure 7D). These results indicate that extended longevity conferred by *unc-62* RNAi involves preventing *ins-7* expression in old age, which results in activation of the insulin signaling pathway and repression of DAF-16/FOXO.

### *unc-62* RNAi extends the lifespan of *glp-1* mutants

It has previously been shown that germline signaling can affect aging, as *glp-1(e2141)* mutants that lack the germline show extended lifespan [41]. We next asked whether the beneficial effects of knockdown of *unc-62* were independent of those from removing a germline by comparing the lifespan of an *unc-62* RNAi*;glp-1(e2141)* double mutant to the lifespan of *glp-1(e2141)* single mutants. We found that *unc-62* RNAi increased the mean lifespan of *glp-1(e2141)* mutants by 28% (*p*<10^−5^), indicating that *unc-62* activity is still detrimental for lifespan in worms lacking a germline (Figure S4C). Previous studies have shown that vitellogenins continue to be expressed in animals lacking a germline [42], [43]. Thus, the lifespan extension by *unc-6*2 RNAi in a *glp-1(e2141)* background may reflect the prevention of vitellogenin accumulation. It may also reflect other downstream effects of *unc-62*, including increased availability of transcriptional resources for intestinal expression and decreased expression of class N genes in old worms.

## Discussion

There are a large number of datasets generated by the modENCODE project that profile targets of transcription factors by ChIP-seq. To identify putative aging regulators, we developed an approach in which we query all transcription factors for those with directly-bound targets that are enriched for altered expression during normal aging. In addition to transcription factors ELT-3 and SKN-1 that have previously been characterized as regulators of aging that can modulate lifespan [10], [21], this approach identified factors that had not previously been linked to gene expression changes that occur during normal aging. The identification of HOX co-factor UNC-62 (Homothorax/hth/Meis) was particularly interesting, as knockdown of *unc-62* in adults significantly extends lifespan [17] but *unc-62* activity is required for embryonic and larval development [13], [14], [15], [16].

We found that *unc-62* acts in the intestine to limit lifespan and that intestinal *unc*-*62(7a)* expression decreases with age. These results suggest that the age-dependent decrease of *unc-62* in the intestine is likely beneficial rather than detrimental for lifespan. With regards to *unc-62* expression, the young state is thus not a model for health as compared to the old state, as this would predict that RNAi of *unc-62* in young worms would shorten their lifespan. In order to better understand why RNAi of *unc-62* results in longer lifespan, we applied a variety of genomics analyses to explore downstream targets of *unc-62* in young and old adults. We identified three downstream effects of *unc-62* activity that can be linked to the aging process: 1) expression of yolk proteins that accumulate with age, 2) generalized effects on transcription of a large number of intestinal genes, and 3) activation of non-intestinal genes in the intestine in old age. The detrimental effects of *unc-62* in adult worms may reflect a combination of these downstream effects, each partially contributing to the overall limitation of lifespan.

### Intestinal UNC-62 and target yolk proteins decline with age

The first mechanism that we identify linking *unc-62* to aging is through direct activation of vitellogenin genes in the intestine. We find that UNC-62 binds to and activates all six vitellogenin genes, and that the decrease in expression of *unc-62(7a)* with age parallels the decline in intestinal expression of vitellogenin genes. These results suggest that the decline with age of UNC-62(7a) contributes to decreased expression of vitellogenins with age. In addition to *unc-62*/Homothorax, we note that it is possible that the age-dependent decline of vitellogenin gene expression may also be due to decreased activity of the GATA transcription factor ELT-2. ELT-2 is a master regulator of intestinal gene expression and also regulates vitellogenin expression through a distinct VPE-2 promoter element [23].

The six vitellogenin genes encode the *C. elegans* yolk proteins, which are utilized for growth and development of progeny. The C. elegans vitellogenin proteins appear to be toxic in old age, as knockdown of *vit-2* and *vit-5* by RNAi results in increased lifespan [39]. During the self-fertile reproductive phase of hermaphrodites (through day five of adulthood [44]), the vitellogenin proteins are removed from the mother through egg-laying. After self-fertile reproduction ends at day five, vitellogenins remain necessary for reproduction by cross-fertilization, which occurs up to day 13 [45]. In the absence of cross-fertilization, vitellogenins accumulate in the body cavity, are subjected to oxidative damage and ultimately become one of the most prevalent proteins in aggregates late in life [5], [46], [47]. Similar to *C. elegans*, vitellogenin proteins in *Drosophila* form aggregates and accumulate oxidative damage in old age [48]. Thus, decreased vitellogenin protein accumulation in the body cavity presents one downstream effect of *unc-62* knockdown that may contribute to extended longevity.

A second mechanism that may link *unc-62* RNAi to the aging process is through allocation of intestinal transcriptional resources. We observe a general increase in expression of intestinal genes upon *unc-62* RNAi. Previous microarray studies have indicated that intestinal genes show a general decrease in expression with age [10], [49], and *unc-62* RNAi has a particularly strong effect on these age-regulated intestinal genes. Thus, while *unc-62(+)* is necessary for intestinal expression of vitellogenins that are produced to provide food for developing embryos, it leads to repression of other intestinal genes. This is reminiscent of the disposable soma theory of aging, in which resource allocation between the soma and the germ-line provides a balance between maternal health and the health and number of progeny [50].

In contrast to the vitellogenins that are directly bound and activated by UNC-62, most of the *unc-62-*repressed intestinal genes are not directly bound by UNC-62. One possible mechanism for how *unc-62* could indirectly repress these genes involves transcriptional resources in the intestine. As vitellogenins are among the most highly-expressed transcripts in the worm genome, silencing of vitellogenins upon *unc-62* RNAi would increase the availability of transcriptional machinery for expression of other genes in the intestine. In this model, *unc-62* would provide a molecular mechanism through which the hermaphrodite intestine allocates transcriptional resources for vitellogenin expression to provide for progeny at the expense of general expression of other intestinal genes that help to maintain somatic health of the mother. The general increase in intestinal expression would not be expected upon RNAi of the vitellogenins themselves, as RNAi would not block vitellogenin gene transcription but rather silence protein production through degradation of vitellogenin mRNA. We do not yet know whether the general increase in intestinal expression that we observe upon *unc-62* RNAi involves only the increased availability of general transcriptional resources, or whether it also requires the activity of specific intestinal regulators (e.g. other transcription factors such as ELT-2).

In addition to vitellogenins, there are many other genes that are bound by UNC-62 in young adults that decrease expression with age in the intestine. However, it is not clear whether age-related decline of these other targets is caused by decreased levels of *unc-62(7a)* or by altered activity of other transcriptional regulators. Using RNA-seq, we found that few of these direct target genes showed changes in expression in *unc-62* RNAi worms, suggesting either that UNC-62 does not regulate their expression or that our RNA-seq method was not sensitive enough to detect changes in their expression. This apparent incongruity between transcription factor binding and either activation or repression is not unique to UNC-62, as many other transcription factors in various species have shown a low overlap between genes that are directly bound and whose expression is responsive to over-expression or knockdown of that transcription factor [51], [52], [53]. Further studies will be required to explore whether UNC-62 binding plays a role in age-regulation of these other genes.

Although our analysis largely focused on the intestine, genetic experiments indicate that activity of *unc-62* in the hypodermis may be important for specifying lifespan. Reduction of *unc-62* activity specifically in the hypodermis extends lifespan, but the targets that are directly activated by UNC-62 in the hypodermis are not known. However, the indirect targets of UNC-62 generally decrease expression with age and include a substantial fraction of hypodermal collagen genes. It remains unclear whether altered collagen expression with age is due to altered activity of *unc-62* itself or additional co-factors.

### An insulin-like gene increases expression and is activated by *unc-62* in the aged intestine

A third way that *unc-62* can modulate longevity is via its role in the aberrant expression of genes in the intestine in old animals. Our analysis identified a set of five class N UNC-62 target genes that were expressed primarily in neuronal tissues during development, but that increased expression in the intestine as animals grow old. One such gene that is decreased in expression in aged intestines upon *unc-62* RNAi treatment is known to contribute to extended longevity, as insulin INS-7 is linked to aging through its role as a signaling molecule in the insulin/IGF signaling pathway [39]. *ins-7* is predominantly expressed in neuronal tissues during development, but increases expression in the intestine with age [36]. In the insulin signaling pathway, insulin binding to the DAF-2 insulin/IGF-1 receptor turns on a signaling cascade that represses the activity of the DAF-16/FOXO transcription factor. *ins-7* has been shown to repress the activity of the insulin signaling pathway, as knockdown of *ins-7* increases lifespan as well as nuclear localization and activity of DAF-16 [36]. We show that *ins-7* is directly activated by UNC-62 in old adult intestines, that intestinal *ins-7* limits lifespan, and that *unc-62* RNAi represses *ins-7* and leads to increased DAF-16 activity in old age. These results indicate that knockdown of *unc-62* allows DAF-16 to remain active and to benefit aged animals. The results showing that *unc-62* is required for expression of *ins-7* in old worms are particularly intriguing in light of the previous finding that intestinal *ins-7* not only represses DAF-16, but that DAF-16 also represses *ins-7* [36]. Thus, the increase of *ins-7* with age appears to trigger a double negative feedback loop that would be prevented by the knockdown of *ins-7* upon *unc-62* RNAi.

If activation of DAF-16 by *unc-62* RNAi in old worms is a major cause for extended longevity, then mutations in *daf-16* should either partially or completely suppress the longevity phenotype of unc-62 RNAi. Our experiments indicated that *daf-16*(*m26*) fully suppresses the longevity phenotype of *unc-62* RNAi animals (Figure 7D). However, a previous study by Curran, *et al.* (2007) using a mutation (*eri-1(mg366)*) that increases sensitivity to RNAi and observed a weaker suppression; in this study, an *eri-1(mg366);daf-16(mgDf47);unc-62 RNAi* triple mutant lived 31% longer than the *eri-1(mg366);daf-16(mgDf47)* double mutant, but 42% shorter than the *eri-1(mg366);unc-62 RNAi* double mutant [17]. It is possible that this discrepancy is due to the use of the *eri-1(mg366)* mutation, as this mutation enables RNAi to affect neuronal tissues that are otherwise insensitive. If so, this could suggest an additional role for *unc-62* mediating lifespan in neuronal tissues.

*unc-62* activates four of the five class N genes that increase expression in the intestine in old age. However, it is unclear whether the increase in expression of these genes with age is caused by changes in UNC-62 activity *per se*. Neither of the *unc-62* splicing isoforms appear to increase expression with age in the intestine; *unc-62(7a)* decreases with age, and we did not observe expression of *unc-62(7b)* (or any other *unc-62* isoforms) in the intestine. Thus, it appears that expression of these genes requires *unc-62* activity, but that their increase in expression with age may be driven by an additional regulator.

The eight class N genes that we tested in this work are a small amount of the total such genes in the genome. Therefore, it is likely that there are many more class N targets with similar behavior that could also contribute to modulation of lifespan by *unc-62*. These results suggest that the old intestine is not simply altered by accumulation of damage or decreased expression of intestinal genes. Instead, the old intestine also appears to differ from the young intestine by an increased expression of class N genes. Because their expression appears to be detrimental, it seems unlikely that age-dependent expression of class N genes is evolutionarily selected. Rather, the onset of class N gene expression in old worms may occur at an age that is beyond the force of natural selection, when there may not be sufficient selective pressure in aged animals to suppress deleterious changes in gene networks.

### Links between development and aging

Recent evidence indicates that transcriptional changes during aging are not only a consequence of damage accumulation, but also reflect altered activity of developmental regulators. Analysis of aging of the human pre-frontal cortex suggests that the majority of age-dependent changes in expression are not unique to aging, but rather mirror both reversals and extensions of developmental expression patterns [54]. In mice, an age-dependent increase in Wnt signaling is associated with a shift from myogenic to fibrogenic lineage phenotypes among mouse muscle stem cells, which is abrogated by the addition of Wnt antagonists [55]. In *C. elegans*, a regulatory circuit in the hypodermal tissue involving three GATA transcription factor genes, *elt-3*, *elt-5*, *and elt-6*, appears to not only modulate lifespan but is also linked to altered expression of target genes with age [10]. Expression of the *elt-5* and *elt-6* GATA transcription factors increase with age, leading to a decrease in expression of the *elt-3* GATA factor. Lifespan is increased in mutants in which *elt-3* expression remains high in old age, suggesting that the age-dependent decrease of *elt-3* expression is detrimental to longevity. As the *elt-5*, *elt-6*, and *elt-3* circuit also plays a key role in specifying the hypodermal fate during development [56], these results suggest that mis-regulation of a key developmental pathway in the hypodermis during aging underlies intrinsic aging of the organism [10].

UNC-62 Homothorax/Meis provides a further example that developmental and reproductive activities of certain transcription factors are not stable in adults, and are not optimized for longer lifespan. To ameliorate or even reverse the aging process, it is not enough to simply prevent damage accumulation. Rather, it may also be necessary to correct developmental regulators responsible for intrinsic changes in the aging transcriptome that limit lifespan.

## Materials and Methods

### ChIP–seq data and analysis

Transcription factor (TF) target identification from chromatin immunoprecipitation followed by high-throughput sequencing (ChIP-seq) data was obtained from the modENCODE consortium (http://submit.modencode.org/submit/public/list *or* http://data.modencode.org/) [18], [20], using datasets available as of 04/30/12. TF Binding sites as well as read density files were mapped to WS220 coordinates using scripts available from WormBase (ftp://ftp.sanger.ac.uk/pub2/wormbase/software/Remap-between-versions/) [57].

All 98 ChIP-seq experiments (profiling targets of 57 transcription factors in one or multiple developmental stages) that contained at least 100 binding sites meeting a *q-*value<10^−5^ significance cutoff were used. ‘Factor-specific’ binding sites of a transcription factor were defined as those that were significantly enriched in the given factor, and had no position within the binding site that was enriched in 9 or more other transcription factors (out of the 57 total) (Figure S1A–S1B). Binding sites were associated with all transcripts (obtained from Wormbase release WS220) if the point of maximal read density within the peak occurred within the gene body, or was less than 3 kb upstream of the annotated transcription start site (for cases where the upstream intergenic distance was less than 3 kb, only the region up to the neighboring gene was used). Genes significantly altered in expression with age (*p*-value<10^−4^) were obtained from [10], with updated mapping of probes to transcripts (http://nemates.org/MA/primers/new_primer_names.html).

Significance of overlap between ChIP-seq targets and various gene lists was determined by a Fisher's Exact test on the 2×2 contingency table using the R statistics program (version 2.11.1), approximated with a Chi-Square test for datasets with expected and observed overlaps greater than 5. *P-*values for Chi-Square tests were obtained using the *Perl* Statistics::Distributions module.

### ChIP–qPCR validation

To validate UNC-62 association with *vit-2* and *vit-6* promoters in adults, ChIP-qPCR was performed on independently generated adult lysates from strain OP600. ChIP was performed using the protocol and GFP antibodies generated by the modENCODE project [18], except for the replacement of sepharose beads with Invitrogen Protein G Dynabeads. qPCR primers were generated that flanked the peak of read density from UNC-62 YA ChIP-seq for the *vit-2* and *vit-6* promoter regions, with three regions bound by hypodermal transcription factor ELT-3 (B0222.8) and pharyngeal and intestinal TF PHA-4 (F32A5.4 and F35G2.1) serving as negative controls. For each, enrichment was determined relative to a non-immunoprecipitated input sample.

To identify sequence motifs in UNC-62 binding sites, the 100 base window surrounding the position of maximum ChIP-seq read density in each UNC-62 binding site was identified as the core binding region. To compare these regions against promoter sequences associated with genes with similar expression and tissue-specificities, 200 bp windows on either side of this core region served as the negative control. A *de novo* motif search for enriched 7-mers was performed using the RSAT program [58], with motif logos generated using WebLogo [59]. To validate the motif, frequencies of the ATTGACA 7-mer sequence were calculated for core and flanking regions, and enrichment calculated by Fisher's Exact test. As an additional control, this analysis was repeated using Fisher-Yates-shuffled core regions as the negative control, yielding similar enrichments.

### RNA–seq profiling of *unc-62* knockdown

RNA-seq quantification upon *unc-62* RNAi was performed using the sterile TJ1060 (*fem-15(b26); spe-9(hc88)*) strain that has previously been used for transcriptome studies in adult worms in order to avoid isolating mRNA from developing embryos [10], [60]. Three independent biological replicate batches of approximately ∼10,000 sterile hermaphrodites were grown at the non-permissive (25°C) temperature until the first day of adulthood, at which point half were placed on plates seeded with *unc-62* RNAi and half on empty vector RNAi. The worms were then grown for 3 days at 20°C, after which RNA was isolated by Trizol extraction followed by phenol-chloroform purification. Poly-A-purified RNA was then isolated using the Qiagen Oligotex Mini kit, and 3′ end enriched RNA-seq libraries were prepared in accordance with the 3′SEQ method. 400 ng of mRNA was heat-sheared for 7.5 minutes at 85°C to obtain 200–500 bp fragments, and barcoded oligo-dT primers were used to generate cDNA for mRNA fragments located at the 3′ end of transcripts. After second strand synthesis, adaptors were ligated to the 5′ end, and PCR primers (including the barcode sequence) were used in order to enable multiplexed sequencing. The three replicate libraries from worms grown on *unc-62* RNAi were then pooled and sequenced in a single flow-cell lane on the Illumina single-end 36 bp HiSeq 2000 platform, with the three libraries from worms grown on control RNAi pooled and sequenced in an additional lane.

A total of ∼71 million and ∼73 million post-filtering reads were obtained for control and *unc-62* RNAi respectively, with roughly equal proportions coming from the three biological replicates (Table S1). Reads with 10 or more consecutive A's or T's were removed as likely amplification artifacts, and reads were then mapped to the Wormbase reference WS215 genome and transcriptome using Bowtie version 0.12.7 [61], using the options “-a –best –strata -v 2” to obtain all genomic position(s) to which the read mapped with the least number mismatches (up to 2 allowed). Greater than 14 million reads for each independent replicate were obtained that mapped uniquely to the sense strand of WS215 annotated transcripts (with more than 85% of post-filter reads mapping to *C. elegans* in each experiment)(Table S1). To determine expression for each transcript in the WS215 Wormbase annotation, we counted the number of reads that uniquely mapped to the sense strand (i.e., they did not map equally well elsewhere in the genome). For genes with multiple annotated isoforms, only the transcript with the highest average expression across the six experiments was used for further analysis.

RNA-seq datasets have been submitted to the Gene Expression Omnibus under series GSE39574 (samples GSM971870-GSM971875).

### Significance analysis by rank products

Identification of transcripts with consistently altered expression in all three biological replicates was patterned on the Rank Product method previously described for microarray analysis [27]. To avoid technical noise among transcripts with low expression, the subset of 7333 genes for which 10 or more sequencing reads were observed either in all three empty vector libraries, or all three *unc-62* RNAi libraries, was obtained, and a pseudocount of one read was added to each gene in each condition. For each of the three biological replicates, genes were then ranked based on the observed expression fold-change between *unc-62* RNAi and control. The rank product for each gene was then calculated as the product of that gene's ranks in each of the three replicate experiments. Repeating this approach on10,000 random permutations of the ranks for each of the three experiments converted these rank product values to E-values and Percent False Positive (PFP) values as previously described [27]. At a PFP cutoff of 10%, 116 genes with decreased expression and 69 genes with increased expression upon *unc-62* RNAi were obtained. Three genes that met this false-positive criteria did not change consistently across the three biological replicates (i.e., were not in the top third of fold-changes in each replicate) and were discarded, yielding 115 genes decreased and 67 genes increased upon *unc-62* RNAi.

### Analysis of genes altered in expression upon *unc-62* RNAi

Significantly increased and decreased genes upon *unc-62* RNAi were compared against various gene lists as described. For all analyses, genes were discarded if they were not present in the 7333 genes with reliable expression in RNA-seq experiments, or were not assayed in the published dataset (as they were either absent on the microarray or were not yet annotated). For analysis of genes that decrease upon *unc-62* RNAi, a dataset of genes with enriched expression in hypodermis (during L3-L4 larval stages) was used [34], and collagen genes were annotated as those belonging to NCBI KOG3544. For analysis of genes that increase upon *unc-62* RNAi, intestine-enriched genes were defined as those with significantly enriched expression in intestine relative to whole-worm controls from experiments profiling intestinal gene expression in late embryos [34], L2 larvae [34], L4 larvae [33], or adults [23]. Genes that decrease expression with age were obtained from Budovskaya, *et al.* [10]. Kolmogorov-Smirnov tests were performed in MATLAB (version R2010b).

### Intestine dissection

Intestine dissections were performed as described by McGhee, *et al*. (2007) [23] with slight modifications. Adult worms were placed in ∼10 uL of buffer containing 100 µl of PBS–EDTA–ATA (125 mM NaCl, 16.6 mM Na_2_HPO_4_, 8.4 mM NaH_2_PO_4_, 0.1 mM EDTA, 1 mM aurin tricarboxylic acid), supplemented with 0.1 uL of Superasin. 27G1/2 needles were used to cut below the pharyngeal bulb, and intestines were allowed to extrude for ∼2–3 minutes. Single intestines were then isolated with a pulled capillary tube, washed 3 times with PBS-EDTA-ATA-Superasin, and placed in Buffer RLT Plus + Beta-mercaptoethanol (Qiagen RNeasy Plus Micro Kit). RNA was isolated from batches of approximately 100 intestines using the RNeasy Plus Micro Kit. RNA quality for one young and one old sample was validated using the RNA 6000 Pico assay on the Bioanalyzer 2100 (Agilent), obtaining RNA integrity numbers of 10 and 9.6 respectively.

To identify class N (neuronal-enriched) young adult UNC-62 targets, we obtained twenty lists of genes with specific or enriched expression in either specific neuronal subtypes or in all neurons [34], [62], [63], [64], [65]. To determine whether UNC-62 binding in young adults correlated with altered expression in intestines with age, a negative control set of genes with identical expression criteria were identified that were associated with UNC-62 binding sites in L2 or L3 but not YA. The initial experiment compared a pair of samples composed of ∼100 intestines from young (day 1) or old (day 15) worms. Transcripts that were undetectable or did not show reliable signal across qPCR technical replicates in young intestines were discarded, as it cannot be determined whether these represented failed primer pairs or if these transcripts are simply never expressed in intestines at levels reliably detected by qPCR. Screening of 9 class N and 31 control genes yielded 7 class N and 6 control genes that were reliably detected in both young and old intestines, with an additional 1 class N and 4 control genes that (presumably due to age down-regulation) were detected in young but not old intestines. For these 5, a Ct value of 40 was used as an upper bound estimate of old adult expression. For each, expression was calculated relative to previously described control transcript *tbb-2* (identical results were observed using alternative control *let-70*) [66]. Normalization was performed as previously described [67].

Further experiments to test the effect of *unc-62* RNAi were performed on two biological replicates of ∼100 intestines for young control (day 1), old control (day 15 grown on empty vector RNAi), and old *unc-62* knockdown (day 15 grown on *unc-62* RNAi) respectively. For old samples, worms were grown on normal (OP50) *E. coli* until day 1 of adulthood, and then shifted to RNAi bacteria (as described for lifespan experiments). For analysis using these samples, transcript abundance was calculated using a 256-fold dilution curve performed on cDNA prepared from whole-worms. Technical triplicates of each qPCR reaction were performed.

### Strains

To generate the *unc-62:GFP* translational reporter, fosmid WRM061dC01 was obtained from Geneservice, and an eGFP tag was inserted at the C-terminus of *unc-62* by recombineering (Figure 1C) [68]. This reporter was integrated by biolistic bombardment, yielding strain OP600 (*unc-119(ed3);Is[unc-62:GFP;unc-119(+)]*. In order to better visualize intestinal expression, this strain was crossed with the *glo-4(ok623)* strain that lacks gut autofluorescence, and *Is[unc-62:GFP;unc-119(+)];glo-4(ok623)* worms were isolated in the F2 generation (strain SD1897).

To generate isoform-specific reporters for *unc-62(7a)* and *unc-62(7b)*, a single base in either exon 7a or 7b was mutated to generate an in-frame stop codon (Figure 2A). This approach required the insertion of a Kanamycin cassette (on the reverse strand) in the constitutively spliced intron between exons 8 and 9 to aid selection for the mutated variant. Biolistic bombardment was used to integrate these fosmids, yielding *Is[unc-62(7a):GFP;unc-119(+)];unc-119(ed3)* (strain OP601) and *Is[unc-62(7b):GFP;unc-119(+)];unc-119(ed3)* (strain OP602) worms. These strains were similarly crossed to *glo-4(ok623)* worms to generate *Is[unc-62(7a):GFP;unc-119(+)];glo-4(ok623)* (strain SD1890) and *Is[unc-62(7b):GFP;glo-4(ok623)* (strain SD1898) which were used for fluorescence measurements.

To determine whether the full-length translational fusion was functional, *unc-119(ed3); Is[unc-62:GFP;unc-119(+)]* was crossed into *unc-62(s472)* animals, and *unc-62(s472); Is[unc-62:GFP;unc-119(+)]* worms were isolated in the F2 generation using a PCR screening approach, yielding healthy strain SD1880. To further validate wild-type function, the full *unc-62:GFP* as well as the isoform-specific *unc-62(7b):GFP* fusions were crossed into *unc-62(e644)* worms containing a loss of function mutation in exon 7b, yielding (*unc-62:GFP;unc-62(e644)*(strain SD1887) and *unc-62(7b):GFP;unc-62(e644)*(strain SD1879) that were isolated by PCR screening of the F2 generation (using allele-specific oligonucleotides targeting the *e644* mutation). Both of these rescued strains have viable phenotypes.

Five tissue-specific RNAi strains from previous publications were used to test the tissue of action for *unc-62*: intestine-specific RNAi strain OLB11, hypodermal-specific RNAi strain NR222, muscle-specific RNAi strain NR350, neuronal- and vulval precursor cell-specific RNAi strain NK742, and uterine-specific RNAi strain NK640. To validate tissue-specific knockdown for intestine-specific and hypodermal-specific strains, these strains were crossed with the *unc-62:GFP* reporter, yielding SD1954 (*Is[unc-62:GFP; unc-119(+)]; rde-1(ne219); Is[lin-26p:rde-1; lin-26p:nls:GFP; rol-6(su1006)]*) and SD1955 (*Is[unc-62:GFP; unc-119(+)]; rde-1(ne219); Is[elt-2p:rde-1; rol-6(su1006)]*). These worms were then placed on control or GFP RNAi as L1 larvae, and imaged as L4 larvae. To assay the effect of *unc-62* RNAi on *daf-16* activity, we used a previously-described reporter that expresses cytoplasmic GFP from the *sod-3* promoter [69].

### Lifespan analysis

General *C. elegans* techniques as well as lifespan experiments were performed as previously described [10]. Unless otherwise noted, lifespan experiments were performed by placing adult worms (with visible eggs) on NGM plates containing 30 uM 5-fluoro-2′-deoxyuridine (FUDR). Deaths before day 7 of adulthood were censured. Log-rank tests were performed using OASIS [70].

### RNAi experiments

RNAi clones used were obtained from the Ahringer RNAi library [71] and sequenced to verify proper insertions. To generate RNAi against exons 7a and 7b of *unc-62*, these exons (as well as ∼50 bp of flanking introns) were PCR amplified and inserted into the L4440 vector at the EcoRV restriction site, and transformed into HT115(de3) *E. coli*. RNAi knockdown experiments in adults (including lifespan experiments) were performed on NGM plates supplemented with 30 uM FUDR, 100 ug/mL Ampicillin, and 2 mM IPTG to induce dsRNA expression.

## Supporting Information

Dataset S1 Rank products analysis of *unc-62* RNAi and control RNA-seq experiments. Rank products analysis is included for all 7333 genes with 10 or more reads observed in all three control, or all three *unc-62* RNAi, RNA-seq replicates. Each gene was ranked in each biological replicate experiment according to the observed fold-change in *unc-62* RNAi as compared to control. The product of ranks across three biological replicates was used to determine the false-positive rate for observing such reproducible fold-change (see Figure S5 and Methods). The first worksheet includes all genes, whereas the following two worksheets include only those genes reproducibly activated or repressed by *unc-62* at a 10% false positive rate (see Methods).
(ZIP)

Figure S1 Examples of factor-specific and non-specific UNC-62 targets. (A–B) Examples of factor-specific and redundant UNC-62 binding sites are shown in genome browser snapshots. Genes are indicated in blue at the top, with exons (boxes) and introns (lines) indicated. Boxes below indicate all regions significantly enriched (*q-*value<10^−5^) in 98 ChIP-seq datasets generated by the modENCODE consortium, with UNC-62 young adult binding sites in red and all other binding sites in black. (A) An UNC-62 binding site in young adults proximal to *vit-2* is factor-specific; the binding site is not significantly enriched in other ChIP-seq experiments. We defined factor-specific targets as those that are significantly enriched in nine or less transcription factors profiled. (B) An UNC-62 young adult binding site in the *rpl-15* promoter is not factor-specific, as many other transcription factors also bind to that region.
(TIF)

Figure S2 UNC-62(7a) expression decreases with age. (A) UNC-62(7a):GFP fluorescence was quantified in the first pair of intestinal nuclei at various ages. Bar height indicates average fluorescence observed per intestinal nuclei for 32–44 worms quantified at each age, with error bars indicating standard error of the mean. We validated these results in an independent sample (right). In all three cases, day 1 adults had significantly higher expression than day 3, 8, or 12 (each *p*<10^−4^ by Student's t-test). (B) *unc-62(7a)* mRNA decreases by qRT-PCR. RNA from ∼75 day 2 and day 8 adult worms (grown on empty vector RNAi) was purified, and qPCR was performed using primers specific to exon 7a of *unc-62*. Each bar indicates average expression from a biological replicate, and error bars indicate the standard deviation among two qPCR technical replicates. For each, expression was first normalized to an *htz-1* control, and then calculated relative to *unc-62(7a)* at day 2 of adulthood.
(TIF)

Figure S3 Tissue-specific RNAi fluorescence validation. To verify that the intestine- and hypodermal-specific RNAi strains function as intended, we crossed in the *unc-62:GFP* reporter. Worms were then placed on control and GFP RNAi as L1 larvae, and imaged as L4 larvae. (A) GFP RNAi in intestine-specific RNAi strain SD1855 (generated from strain OLB11) does not affect hypodermal (hyp.) or neuronal/ventral nerve cord (vnc) expression of *unc-62:GFP*, but decreases *unc-62:GFP* expression in the intestine (int). Although most intestinal cells showed complete loss of *unc-62:GFP* expression, some nuclei still had visible *unc-62:GFP* expression (as indicated). (B) GFP RNAi in hypodermal-specific RNAi strain SD1854 (generated from strain NR222) does not affect intestinal or neuronal/ventral nerve cord expression of *unc-62:GFP*. However, hypodermal expression of *unc-62:GFP* was no longer visible.
(TIF)

Figure S4 Lifespan analysis of *unc-62*. (A) Wild-type (N2) worms were grown on control bacteria, and then shifted to *unc-62* at various days of adulthood to determine the time of effect of *unc-62* knockdown. *unc-62* RNAi significantly increased lifespan when begun at days 1, 3, or 5 of adulthood (*p*<10^−5^, *p*<10^−5^, and *p = *0.0003 respectively). RNAi of *unc-62* beginning at days 7 or 9 of adulthood did not significantly extend lifespan (*p = *0.15 and 0.63 respectively). As the rate of feeding declines significantly with age, we do not know whether the lack of effect observed at day seven indicates that *unc-62* no longer limits lifespan or simply reflects the weakened effect of RNAi by feeding. (B) Lifespan of knockdown of *unc-62*, *ins-7*, and combined RNAi targeting both *unc-62* and *ins-7* was performed in a strain expressing *rde-1* from the *elt-2* intestinal promoter (strain OLB11). Intestine-specific *ins-7* knockdown extends lifespan 8.3% (*p*<10^−5^ by log-rank test). Combined RNAi targeting both *unc-62* and *ins-7* showed a 6.1% increase over *unc-62* alone (*p = *0.0347), suggesting that *unc-62* RNAi by itself may not completely silence *ins-7* expression in the intestine. (C) RNAi of *unc-62* in germ-line mutant *glp-1(e2141)* worms significantly extends lifespan (28%, *p*<10^−5^). To remove the germ-line, *glp-1(e2141)* worms were grown at the restrictive temperature (25°C) until the first day of adulthood, and then shifted to 20°C.
(TIF)

Figure S5 Rank products method for identifying differential expression in biologically replicated RNA-seq experiments. In order to identify genes that were consistently increased or decreased in expression upon *unc-62* RNAi, we developed an analysis method based on the Rank Products method previously described for microarray analysis [27]. (A) We performed three independent experiments in which we fed worms either *unc-62* RNAi or control bacteria, isolated mRNA and generated RNA-seq libraries, and sequenced these libraries on the Illumina HiSeq platform. For analysis, we discarded genes that were not covered by at least 10 sequencing reads in either controls or *unc-62* knockdown across all three replicates (red arrows). (B) For each gene in each replicated experiment, the fold-change was calculated between experimental (*unc-62* RNAi) and control. These fold-changes were then ranked for each replicate. (C–D) A rank product (RP) was then calculated for each gene as the product of the fold-change ranks in each replicate, and converted to a probability. The ranks were then permuted 10,000 times to generate the expected number (E-value) of genes observed with a probability less than or equal to the observed RP. This E-value could be converted to a false-positive rate by dividing by the number of genes actually observed to have such a RP probability. (C) Genes were ranked by increasing fold-change upon *unc-62* RNAi (starting with the most down-regulated gene) to calculate a false-positive rate for decreased expression upon *unc-62* RNAi. (D) Genes were ranked by decreasing fold-change upon *unc-62* RNAi (starting with the most up-regulated gene) to calculate a false-positive rate for increase expression upon *unc-62* RNAi.
(TIF)

Figure S6 ChIP-qPCR validates UNC-62 association at *vit-2* and *vit-6* promoters in young adult worms. OP600 (*unc-62:*GFP) worms were grown on peptone plates until adulthood, at which point they were switched to NGM plates containing 30 µM FUDR until day 4 of adulthood. ChIP was performed using the protocol from the modENCODE consortium [18], and qPCR was performed for *vit-2* and *vit-6* promoter regions as well as three regions bound by other transcription factors but not UNC-62 (B0222.8, F32A5.4, and F35G2.1). For each, fold-enrichment was calculated by comparing first to a non-immunoprecipitated input sample, and then to the first control promoter region (B0222.8). Bars indicate mean, and error bars indicate standard deviation from technical triplicate qPCR measurements.
(TIF)

Figure S7 *De novo* motif searches identify an ATTGACA motif among UNC-62 adult binding sites. (A) Two of the three motifs identified in a *de novo* motif search of UNC-62 young adult binding sites with RSAT [58] contained an ATTGACA motif as the prominent sequence motif. In this analysis, the core 100 nt regions of the UNC-62 factor-specific binding sites (centered upon the point of maximal read density within the peak) were compared to 200 nt regions flanking this core region. Motif 2 does not contain the conserved TGACA Homothorax motif [31], but instead resembles the GATA binding site of master intestinal regulator ELT-2 [23]. (B) The ATTGACA 7-mer is the most significantly enriched 7-mer sequence among UNC-62 binding sites. (top) The fold-enrichment of all 7-mers was compared (top) in the core region as compared to the flanking regions of UNC-62 binding sites, or (b) the core region as compared to core region sequences randomly shuffled 100 times (using Fisher-Yates shuffling). Enrichment *p-*values were determined by Fisher's Exact test.
(TIF)

Figure S8 Intestine-enriched genes are uniquely shifted towards higher expression upon *unc-62* RNAi. Histograms indicate the percent of these gene sets with indicated fold-changes in expression (shown in 0.1 (log2) increments) in RNA-seq of adult worms exposed to *unc-62* RNAi. Datasets were obtained from previous publications for genes with enriched expression in (A) muscle cells [33], [34], [72], (B) neuronal cells [34], [62], [63], [64], [65], and (C) intestinal cells [23], [33], [34], as well as (D) the subset of intestine-enriched genes that also decline in expression with age [10]. Intestine-enriched and age-decreased, intestine-enriched genes are significantly shifted towards increased expression upon *unc-62* RNAi (*p-*value<10^−20^ by Kolmogorov-Smirnov test), whereas muscle- and neuronal-enriched genes are not (*p*>0.01 by Kolmogorov-Smirnov test).
(TIF)

Figure S9 Dissected intestines are enriched for intestine-specific transcripts and depleted for non-intestinal transcripts. Transcripts include two ubiquitously expressed reference genes (*let-70* and *tbb-2*), two intestine-specific positive controls (*ges-1* and *vit-6*), and four non-intestinal genes: pharynx-specific *myo-2*, germline-specific *pie-1*, neuronal *unc-119*, and hypodermis and gonad-expressed *lin-26*. RNA was isolated from micro-dissected intestines as described in Methods, and reverse transcribed into cDNA for quantification by qPCR. Bars indicate ΔΔC_t_ values are calculated as (Ct_intestine,gene x_-Ct_whole worm, gene x_)- (Ct_intestine,M7.1_-Ct_whole worm, M7.1_), using reference M7.1. All Ct values are averaged from triplicate technical replicates. Error bars indicate standard deviation of triplicate technical replicates. * indicates genes that were undetectable in two of three technical replicates; for these, the expression in the single amplified replicate was used to estimate expression. ** indicates genes that did not amplify from the indicated sample; for these experiments, an upper bound estimate of expression of 40 cycles was used.
(TIF)

Figure S10 Both *tsp-1* and *tsp-2* are activated by UNC-62 in old adult intestines. An UNC-62 young adult binding site (indicated by the red box) located just upstream of *tsp-2* was initially associated with *tsp-1*, as the point of maximal read density was located within the *tsp-1* transcript sequence (Figure 7B). We performed qRT-PCR on RNA isolated from ∼150 micro-dissected intestines to quantify *tsp-2* expression. Similar to *tsp-1*, we found that *tsp-2* expression is induced in old adults but not induced to the same degree in old adults fed *unc-62* RNAi. Bars indicate expression in biological replicate experiments, relative to expression in young adults (normalized to control gene *tbb-2*). Error bars indicate standard deviation from triplicate technical replicates.
(TIF)

Table S1 RNA-seq experiment summary information. Values indicate number of reads meeting the stated criteria observed from RNA-seq experiments performed on three biological replicates of day four adult worms grown either on empty vector control, or *unc-62* RNAi. “Post-quality filtering” indicates reads that did not contain a stretch of ten or more consecutive A or T nucleotides (which were discarded as likely artifacts from the reverse transcription oligonucleotide). Reads were mapped to the WS215 genome and annotated transcripts with Bowtie (see Methods), and were then discarded if they mapped with an equal number of mismatches to multiple positions in the genome. To simplify downstream analyses, at the final step only reads mapping to the sense strand of annotated transcripts were used. “Vitellogenin transcripts” indicates the number of reads in each experiment that mapped to the six vitellogenin loci (in this row, reads were kept if they mapped equally well to multiple vitellogenin loci but to no other loci in the genome).
(XLSX)

Table S2 Data for lifespan experiments. Each row in the table describes data from a lifespan experiment described in the text, grouped by experiments performed at the same time. *p*-values were calculated by log-rank test in Oasis [70] as compared to the first listed empty vector control unless otherwise specified. For all experiments, worms were grown on OP50 *E. coli* until the first day of adulthood and then shifted to bacteria expressing the indicated dsRNA. For tissue-specific RNAi experiments, the Tissue column indicates the tissue in which the RNAi pathway is active. For time of action experiments, worms were shifted to HT115 (empty vector) bacteria on the first day of adulthood, and then shifted to *unc-62* RNAi bacteria at the indicated day for the remainder of lifespan.
(XLSX)

Table S3 Genes bound by UNC-62 and differentially regulated upon *unc-62* RNAi. Each row indicates a gene associated with an UNC-62 binding site in day four young adults that was identified to be significantly altered upon *unc-62* RNAi in the RNA-seq experiment. Fold-change is shown as the average fold-change from the three biological replicates (after each experiment was independently mean-normalized). Distance to TSS indicates the distance from the annotated transcription start site to the point of maximal read density within the ChIP-seq peak. For genes with multiple isoforms, the shortest distance is reported.
(XLSX)
