# Supplementary figures and images for: Roles of the Developmental Regulator unc-62/Homothorax in Limiting Longevity in Caenorhabditis elegans
Source: PLoS Genet. 2013 Feb 28;9(2):e1003325. doi: 10.1371/journal.pgen.1003325 (PMC3585033; doi:10.1371/journal.pgen.1003325)

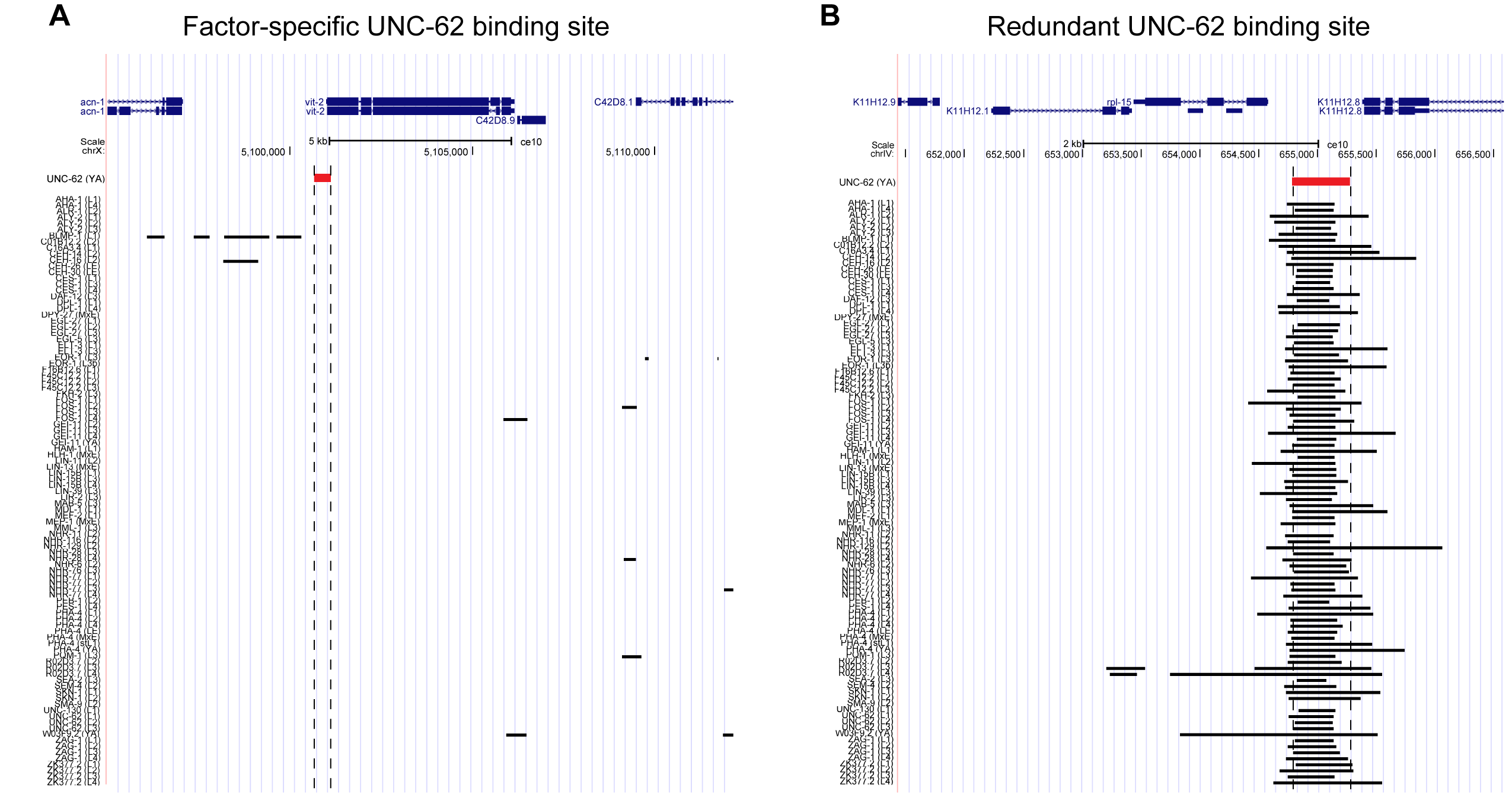

Supplement: Figure S1 — Examples of factor-specific and non-specific UNC-62 targets. (A–B) Examples of factor-specific and redundant UNC-62 binding sites are shown in genome browser snapshots. Genes are indicated in blue at the top, with exons (boxes) and introns (lines) indicated. Boxes below indicate all regions significantly enriched (q-value<10−5) in 98 ChIP-seq datasets generated by the modENCODE consortium, with UNC-62 young adult binding sites in red and all other binding sites in black. (A) An UNC-62 binding site in young adults proximal to vit-2 is factor-specific; the binding site is not significantly enriched in other ChIP-seq experiments. We defined factor-specific targets as those that are significantly enriched in nine or less transcription factors profiled. (B) An UNC-62 young adult binding site in the rpl-15 promoter is not factor-specific, as many other transcription factors also bind to that region. (TIF) [file pgen.1003325.s002.tif]

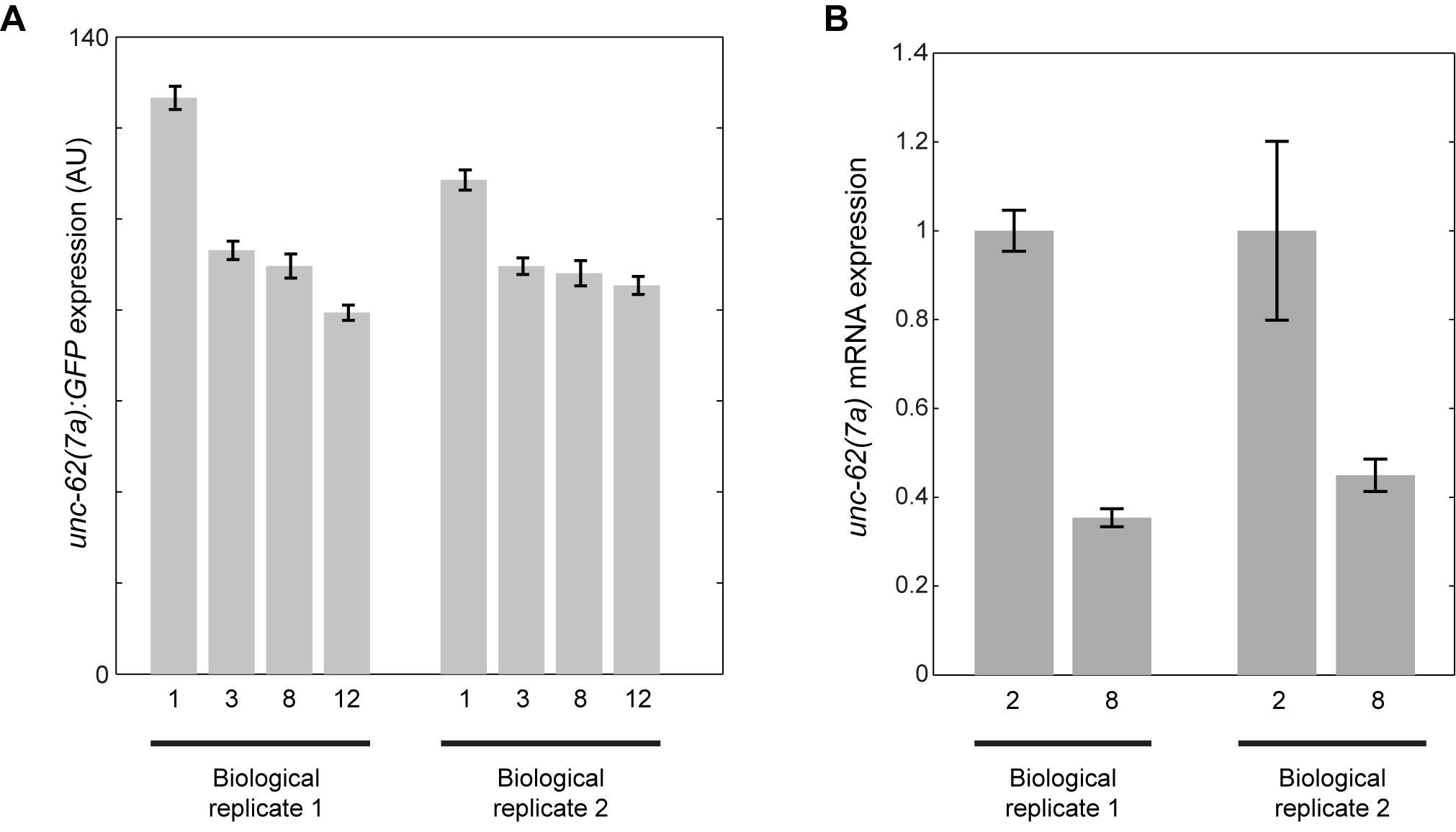

Supplement: Figure S2 — UNC-62(7a) expression decreases with age. (A) UNC-62(7a):GFP fluorescence was quantified in the first pair of intestinal nuclei at various ages. Bar height indicates average fluorescence observed per intestinal nuclei for 32–44 worms quantified at each age, with error bars indicating standard error of the mean. We validated these results in an independent sample (right). In all three cases, day 1 adults had significantly higher expression than day 3, 8, or 12 (each p<10−4 by Student's t-test). (B) unc-62(7a) mRNA decreases by qRT-PCR. RNA from ∼75 day 2 and day 8 adult worms (grown on empty vector RNAi) was purified, and qPCR was performed using primers specific to exon 7a of unc-62. Each bar indicates average expression from a biological replicate, and error bars indicate the standard deviation among two qPCR technical replicates. For each, expression was first normalized to an htz-1 control, and then calculated relative to unc-62(7a) at day 2 of adulthood. (TIF) [file pgen.1003325.s003.tif]

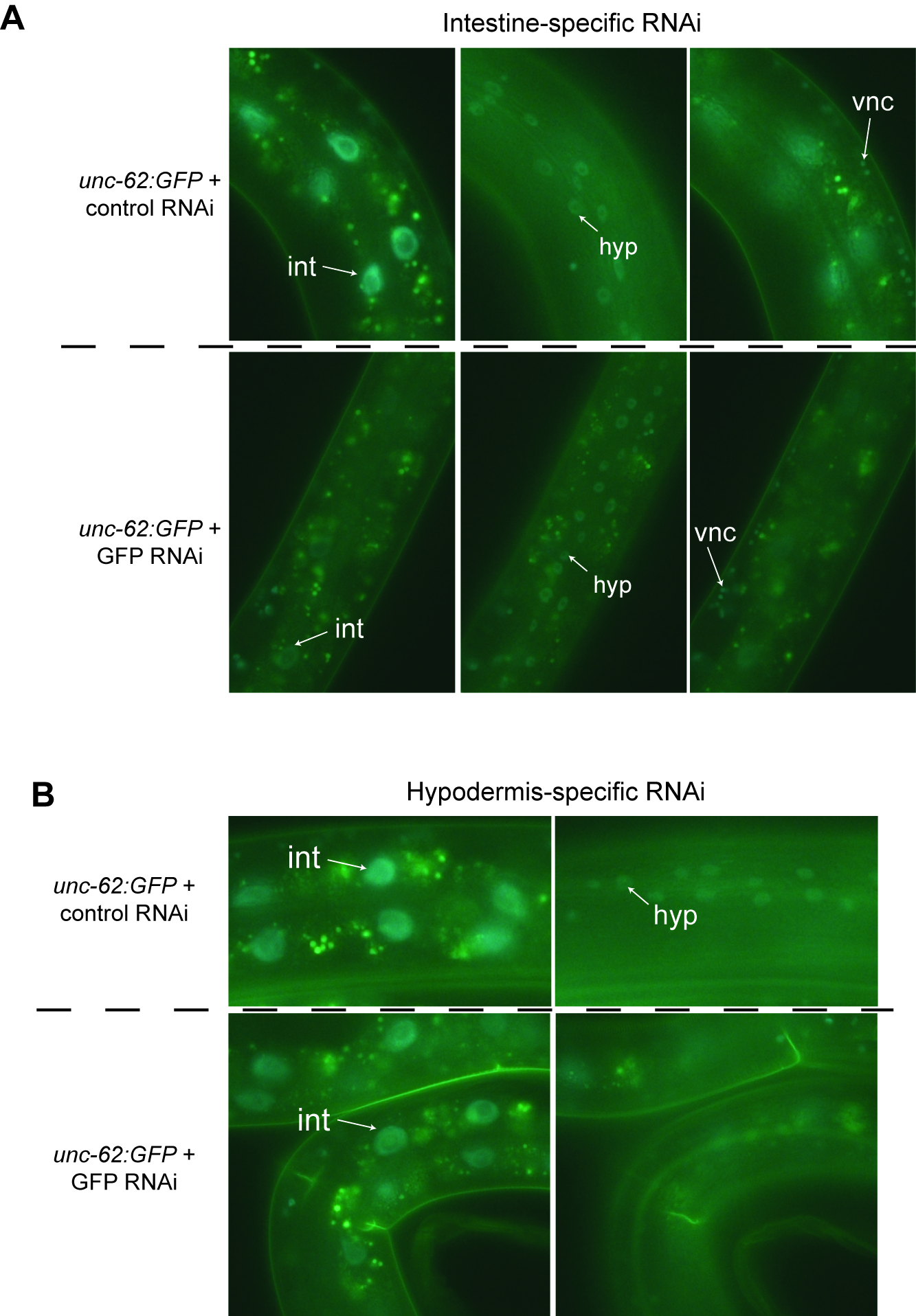

Supplement: Figure S3 — Tissue-specific RNAi fluorescence validation. To verify that the intestine- and hypodermal-specific RNAi strains function as intended, we crossed in the unc-62:GFP reporter. Worms were then placed on control and GFP RNAi as L1 larvae, and imaged as L4 larvae. (A) GFP RNAi in intestine-specific RNAi strain SD1855 (generated from strain OLB11) does not affect hypodermal (hyp.) or neuronal/ventral nerve cord (vnc) expression of unc-62:GFP, but decreases unc-62:GFP expression in the intestine (int). Although most intestinal cells showed complete loss of unc-62:GFP expression, some nuclei still had visible unc-62:GFP expression (as indicated). (B) GFP RNAi in hypodermal-specific RNAi strain SD1854 (generated from strain NR222) does not affect intestinal or neuronal/ventral nerve cord expression of unc-62:GFP. However, hypodermal expression of unc-62:GFP was no longer visible. (TIF) [file pgen.1003325.s004.tif]

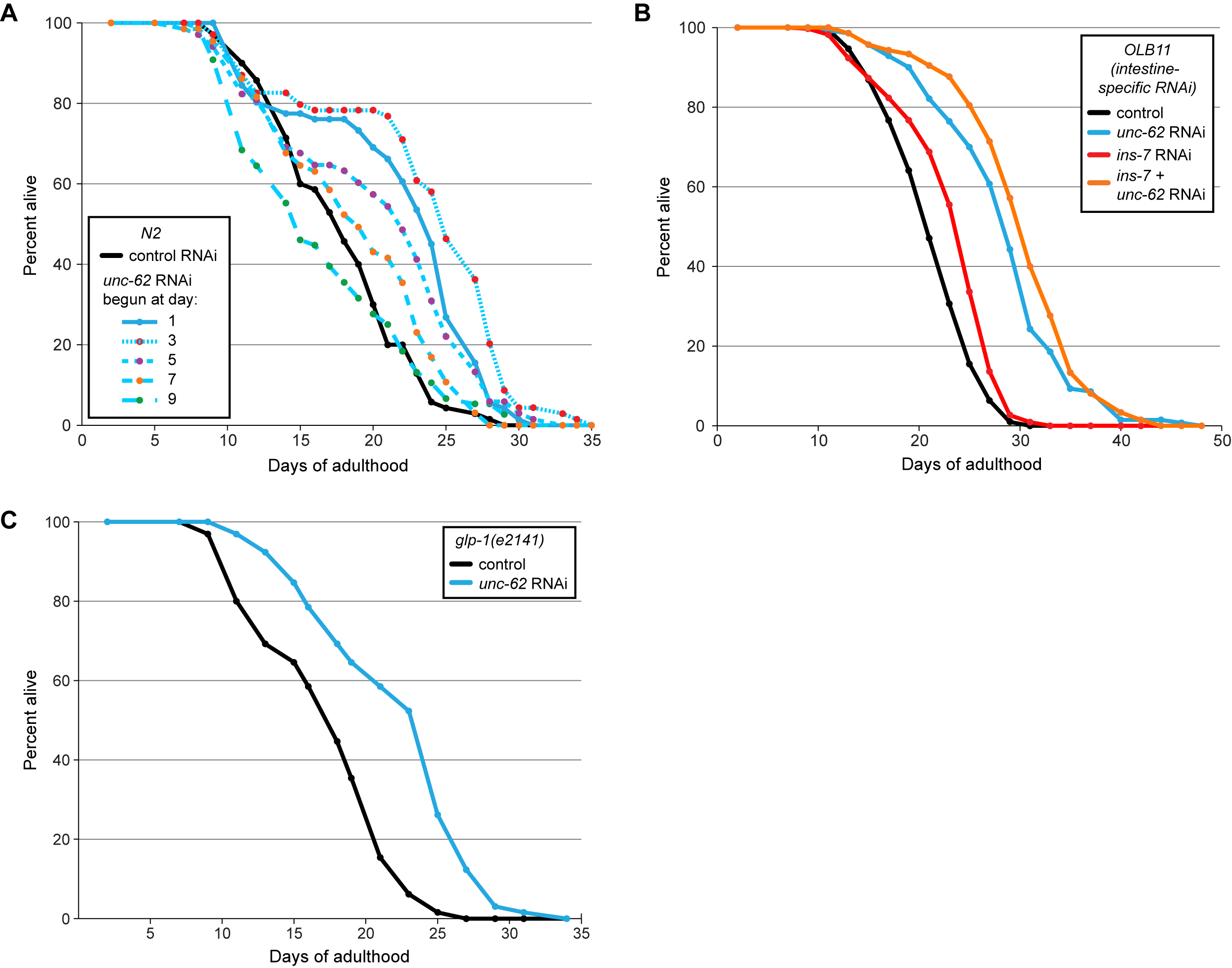

Supplement: Figure S4 — Lifespan analysis of unc-62. (A) Wild-type (N2) worms were grown on control bacteria, and then shifted to unc-62 at various days of adulthood to determine the time of effect of unc-62 knockdown. unc-62 RNAi significantly increased lifespan when begun at days 1, 3, or 5 of adulthood (p<10−5, p<10−5, and p = 0.0003 respectively). RNAi of unc-62 beginning at days 7 or 9 of adulthood did not significantly extend lifespan (p = 0.15 and 0.63 respectively). As the rate of feeding declines significantly with age, we do not know whether the lack of effect observed at day seven indicates that unc-62 no longer limits lifespan or simply reflects the weakened effect of RNAi by feeding. (B) Lifespan of knockdown of unc-62, ins-7, and combined RNAi targeting both unc-62 and ins-7 was performed in a strain expressing rde-1 from the elt-2 intestinal promoter (strain OLB11). Intestine-specific ins-7 knockdown extends lifespan 8.3% (p<10−5 by log-rank test). Combined RNAi targeting both unc-62 and ins-7 showed a 6.1% increase over unc-62 alone (p = 0.0347), suggesting that unc-62 RNAi by itself may not completely silence ins-7 expression in the intestine. (C) RNAi of unc-62 in germ-line mutant glp-1(e2141) worms significantly extends lifespan (28%, p<10−5). To remove the germ-line, glp-1(e2141) worms were grown at the restrictive temperature (25°C) until the first day of adulthood, and then shifted to 20°C. (TIF) [file pgen.1003325.s005.tif]

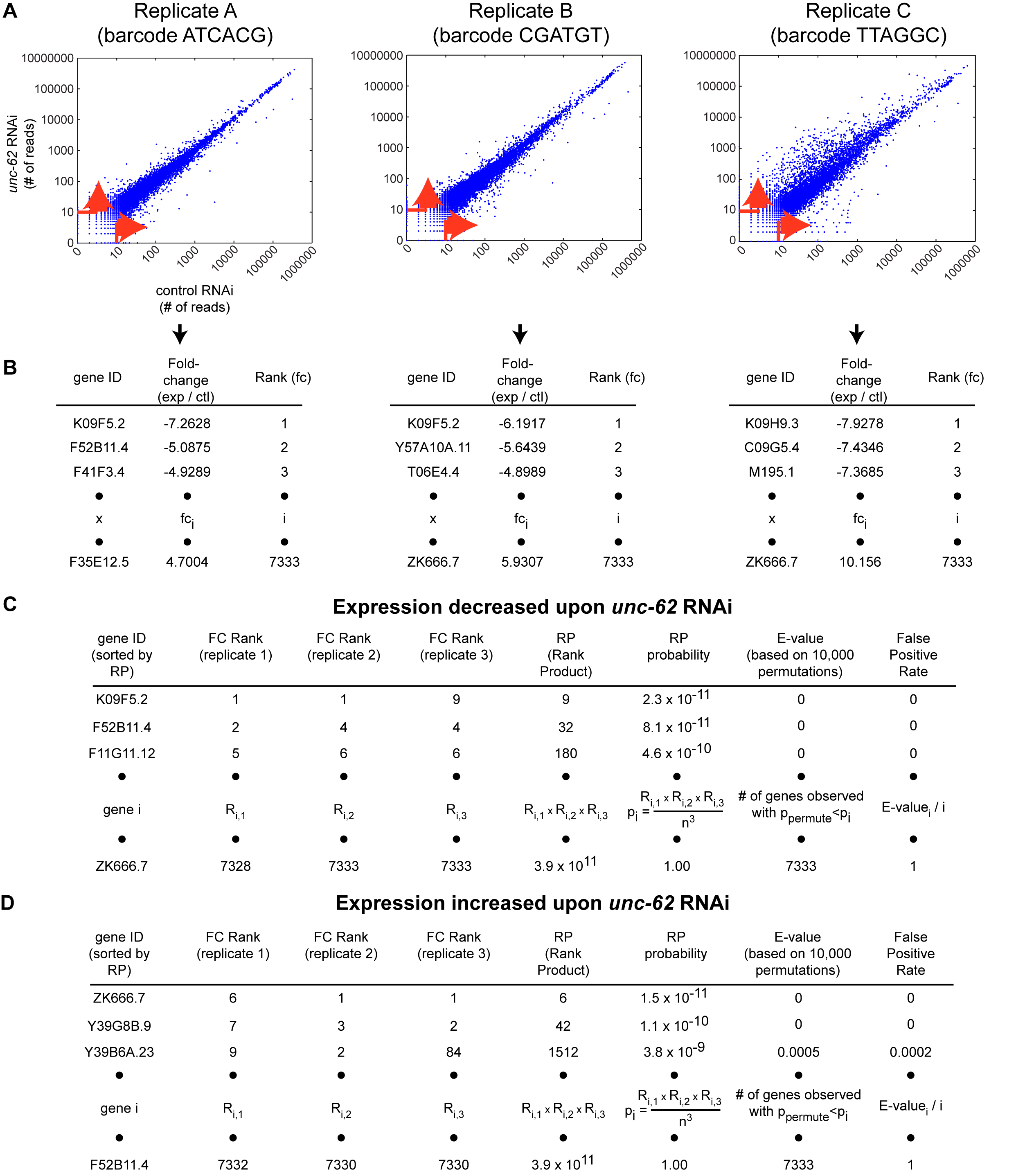

Supplement: Figure S5 — Rank products method for identifying differential expression in biologically replicated RNA-seq experiments. In order to identify genes that were consistently increased or decreased in expression upon unc-62 RNAi, we developed an analysis method based on the Rank Products method previously described for microarray analysis [27]. (A) We performed three independent experiments in which we fed worms either unc-62 RNAi or control bacteria, isolated mRNA and generated RNA-seq libraries, and sequenced these libraries on the Illumina HiSeq platform. For analysis, we discarded genes that were not covered by at least 10 sequencing reads in either controls or unc-62 knockdown across all three replicates (red arrows). (B) For each gene in each replicated experiment, the fold-change was calculated between experimental (unc-62 RNAi) and control. These fold-changes were then ranked for each replicate. (C–D) A rank product (RP) was then calculated for each gene as the product of the fold-change ranks in each replicate, and converted to a probability. The ranks were then permuted 10,000 times to generate the expected number (E-value) of genes observed with a probability less than or equal to the observed RP. This E-value could be converted to a false-positive rate by dividing by the number of genes actually observed to have such a RP probability. (C) Genes were ranked by increasing fold-change upon unc-62 RNAi (starting with the most down-regulated gene) to calculate a false-positive rate for decreased expression upon unc-62 RNAi. (D) Genes were ranked by decreasing fold-change upon unc-62 RNAi (starting with the most up-regulated gene) to calculate a false-positive rate for increase expression upon unc-62 RNAi. (TIF) [file pgen.1003325.s006.tif]

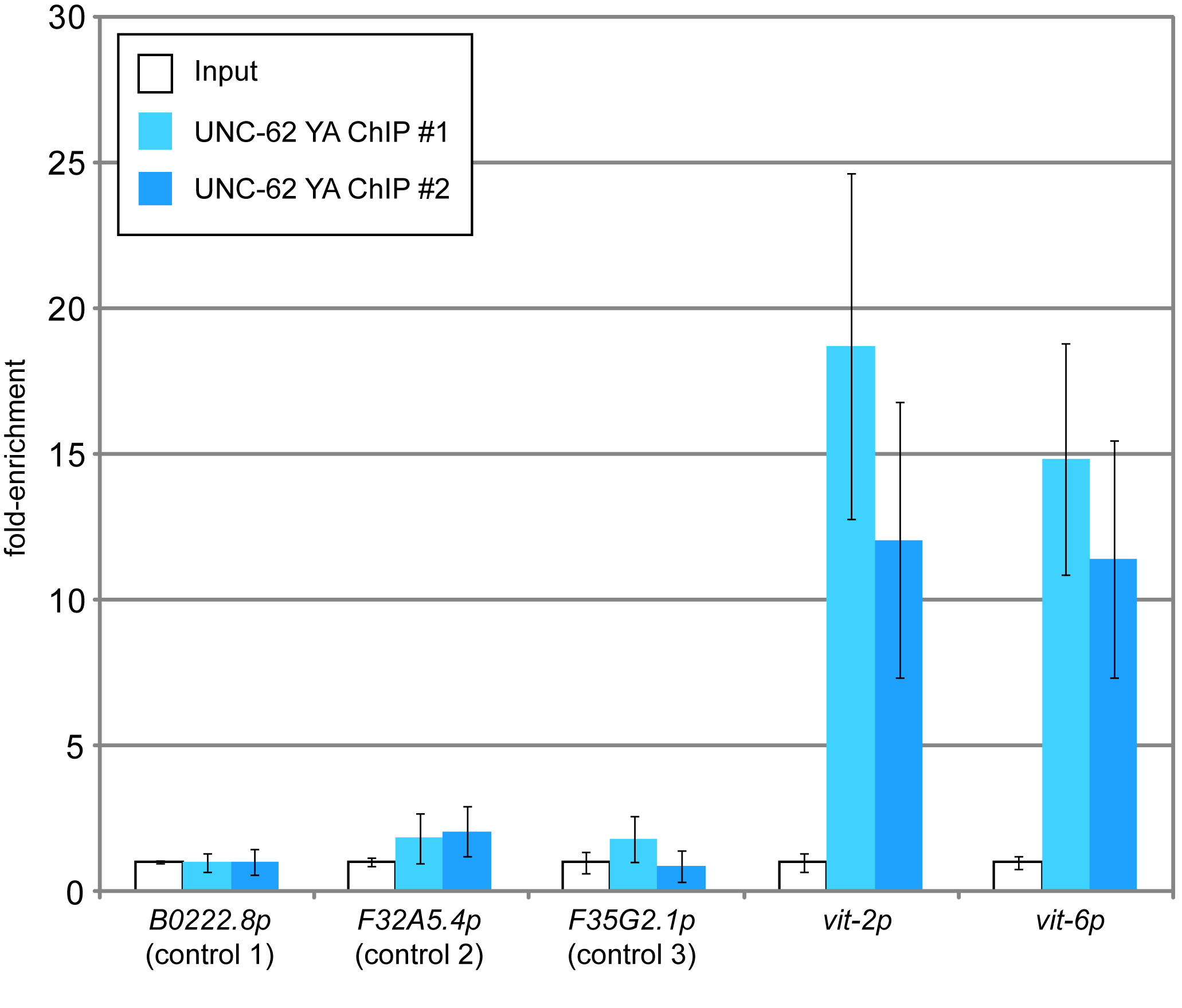

Supplement: Figure S6 — ChIP-qPCR validates UNC-62 association at vit-2 and vit-6 promoters in young adult worms. OP600 (unc-62:GFP) worms were grown on peptone plates until adulthood, at which point they were switched to NGM plates containing 30 µM FUDR until day 4 of adulthood. ChIP was performed using the protocol from the modENCODE consortium [18], and qPCR was performed for vit-2 and vit-6 promoter regions as well as three regions bound by other transcription factors but not UNC-62 (B0222.8, F32A5.4, and F35G2.1). For each, fold-enrichment was calculated by comparing first to a non-immunoprecipitated input sample, and then to the first control promoter region (B0222.8). Bars indicate mean, and error bars indicate standard deviation from technical triplicate qPCR measurements. (TIF) [file pgen.1003325.s007.tif]

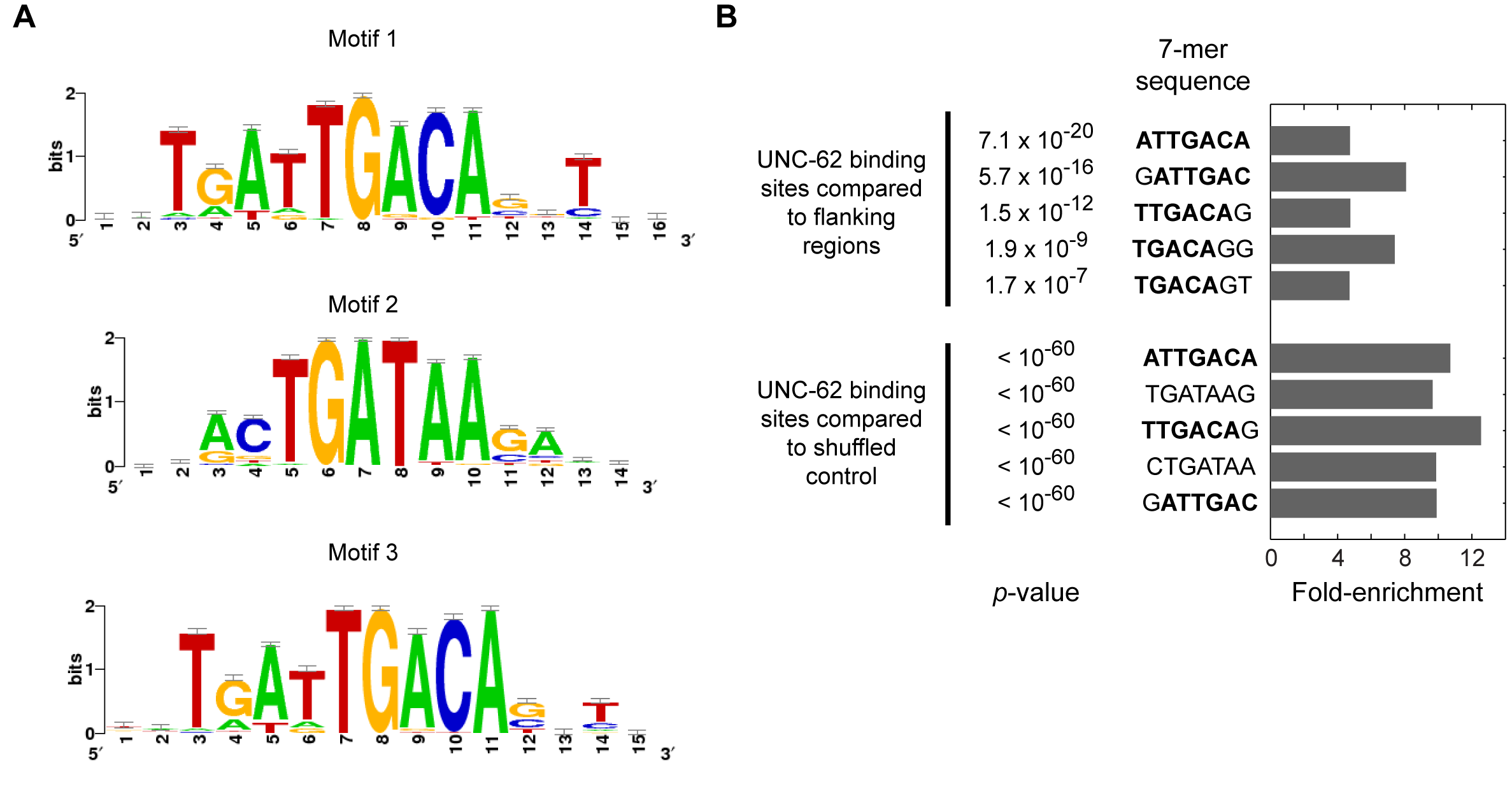

Supplement: Figure S7 — De novo motif searches identify an ATTGACA motif among UNC-62 adult binding sites. (A) Two of the three motifs identified in a de novo motif search of UNC-62 young adult binding sites with RSAT [58] contained an ATTGACA motif as the prominent sequence motif. In this analysis, the core 100 nt regions of the UNC-62 factor-specific binding sites (centered upon the point of maximal read density within the peak) were compared to 200 nt regions flanking this core region. Motif 2 does not contain the conserved TGACA Homothorax motif [31], but instead resembles the GATA binding site of master intestinal regulator ELT-2 [23]. (B) The ATTGACA 7-mer is the most significantly enriched 7-mer sequence among UNC-62 binding sites. (top) The fold-enrichment of all 7-mers was compared (top) in the core region as compared to the flanking regions of UNC-62 binding sites, or (b) the core region as compared to core region sequences randomly shuffled 100 times (using Fisher-Yates shuffling). Enrichment p-values were determined by Fisher's Exact test. (TIF) [file pgen.1003325.s008.tif]

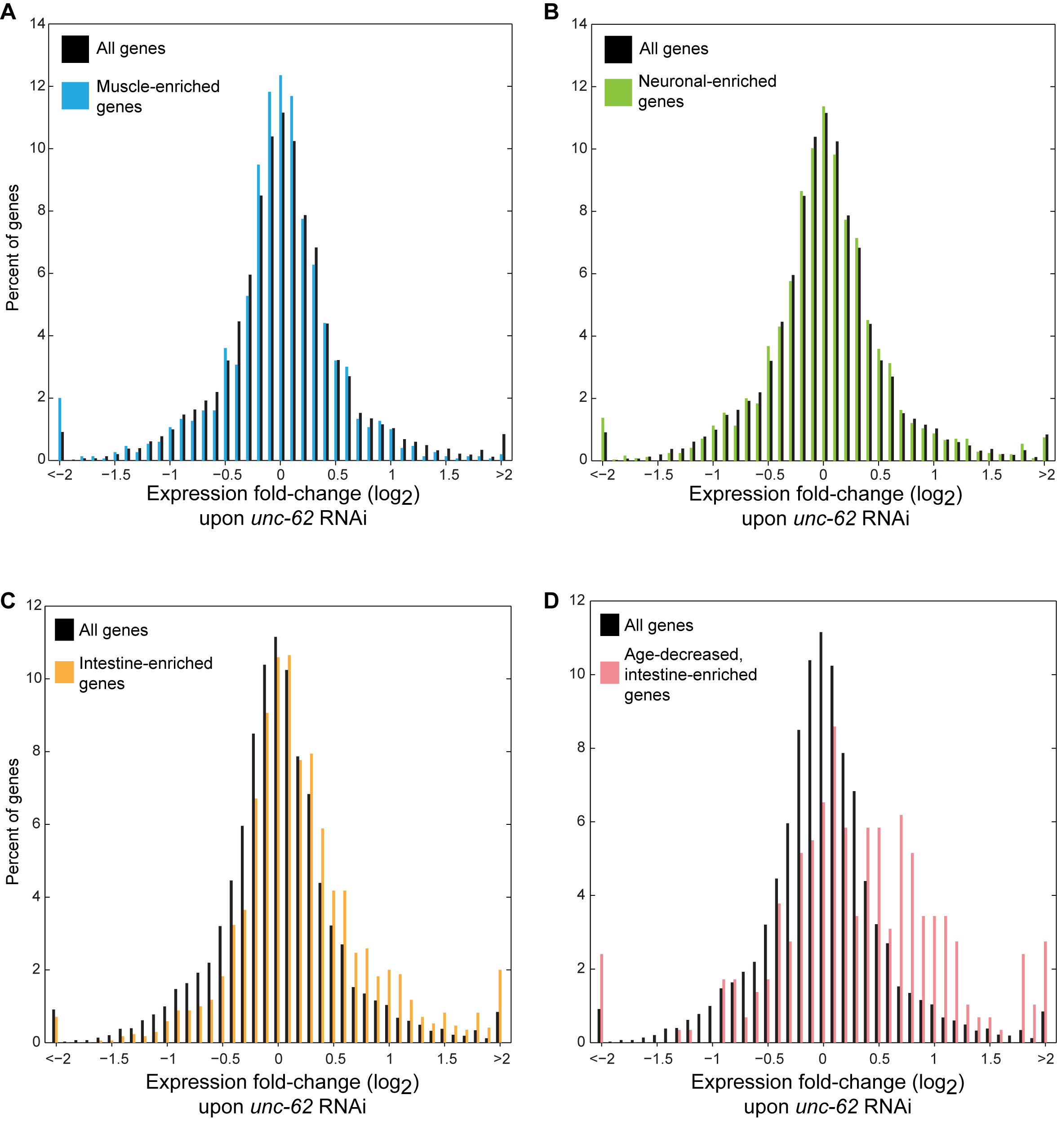

Supplement: Figure S8 — Intestine-enriched genes are uniquely shifted towards higher expression upon unc-62 RNAi. Histograms indicate the percent of these gene sets with indicated fold-changes in expression (shown in 0.1 (log2) increments) in RNA-seq of adult worms exposed to unc-62 RNAi. Datasets were obtained from previous publications for genes with enriched expression in (A) muscle cells [33], [34], [72], (B) neuronal cells [34], [62], [63], [64], [65], and (C) intestinal cells [23], [33], [34], as well as (D) the subset of intestine-enriched genes that also decline in expression with age [10]. Intestine-enriched and age-decreased, intestine-enriched genes are significantly shifted towards increased expression upon unc-62 RNAi (p-value<10−20 by Kolmogorov-Smirnov test), whereas muscle- and neuronal-enriched genes are not (p>0.01 by Kolmogorov-Smirnov test). (TIF) [file pgen.1003325.s009.tif]

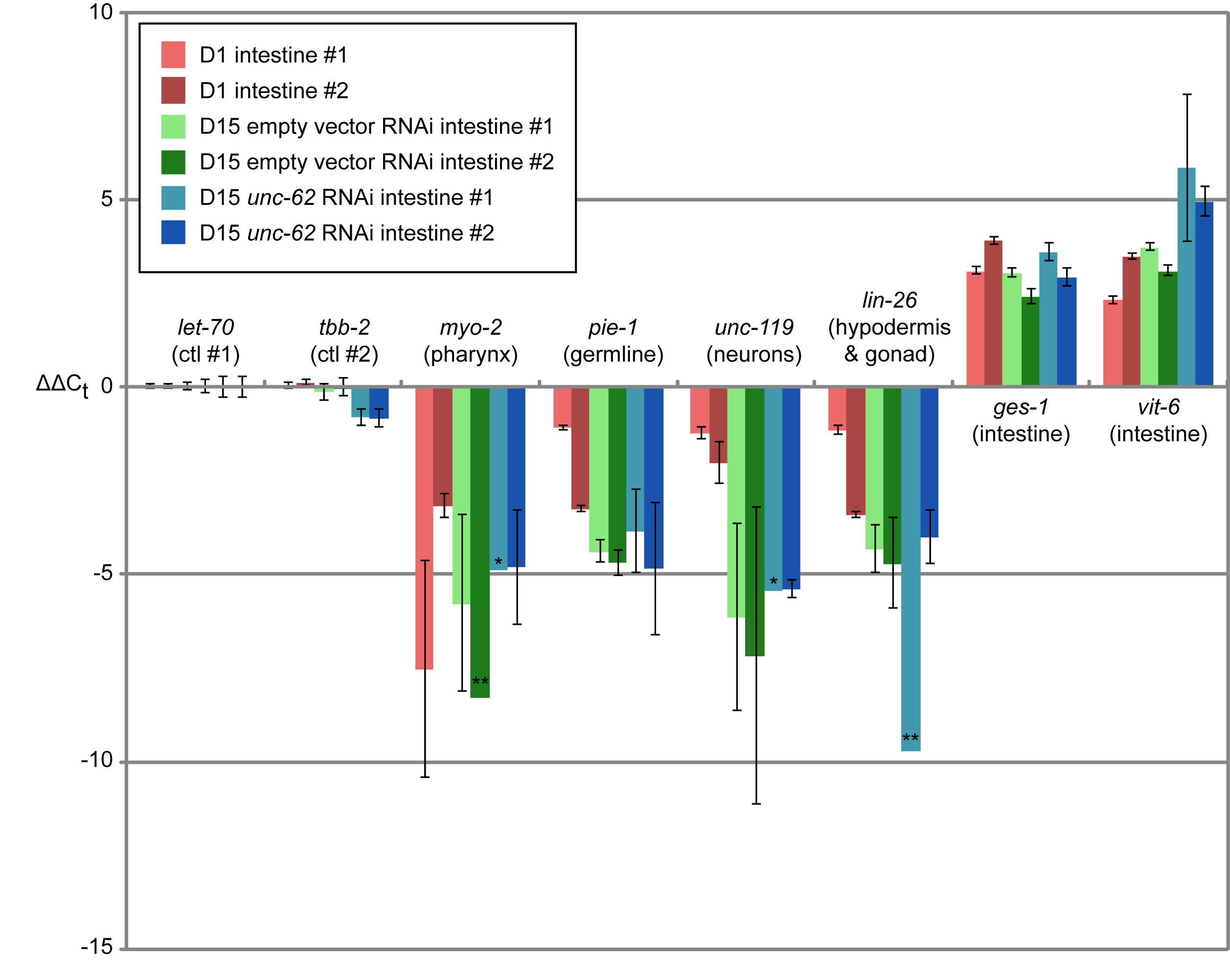

Supplement: Figure S9 — Dissected intestines are enriched for intestine-specific transcripts and depleted for non-intestinal transcripts. Transcripts include two ubiquitously expressed reference genes (let-70 and tbb-2), two intestine-specific positive controls (ges-1 and vit-6), and four non-intestinal genes: pharynx-specific myo-2, germline-specific pie-1, neuronal unc-119, and hypodermis and gonad-expressed lin-26. RNA was isolated from micro-dissected intestines as described in Methods, and reverse transcribed into cDNA for quantification by qPCR. Bars indicate ΔΔCt values are calculated as (Ctintestine,gene x-Ctwhole worm, gene x)- (Ctintestine,M7.1-Ctwhole worm, M7.1), using reference M7.1. All Ct values are averaged from triplicate technical replicates. Error bars indicate standard deviation of triplicate technical replicates. * indicates genes that were undetectable in two of three technical replicates; for these, the expression in the single amplified replicate was used to estimate expression. ** indicates genes that did not amplify from the indicated sample; for these experiments, an upper bound estimate of expression of 40 cycles was used. (TIF) [file pgen.1003325.s010.tif]

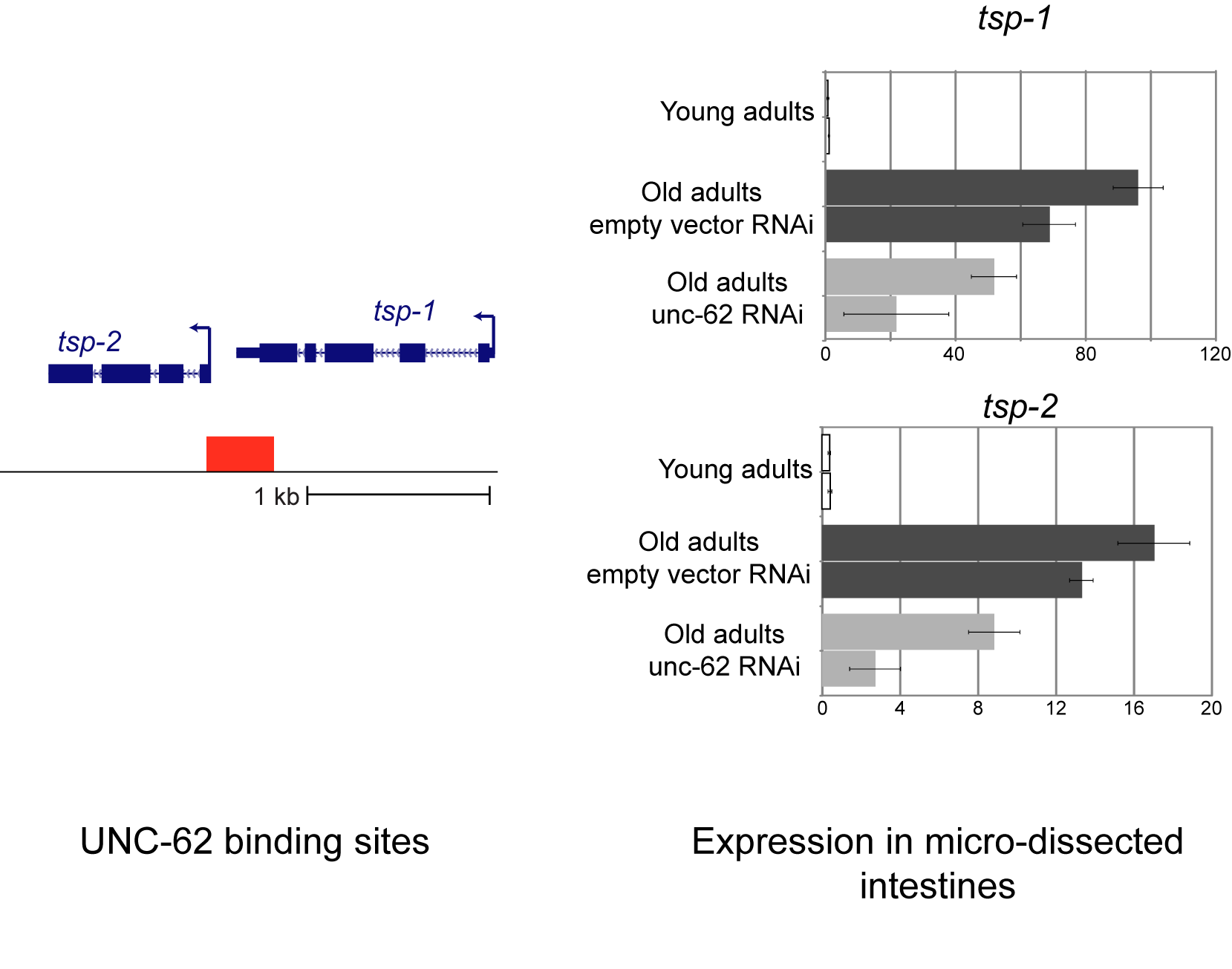

Supplement: Figure S10 — Both tsp-1 and tsp-2 are activated by UNC-62 in old adult intestines. An UNC-62 young adult binding site (indicated by the red box) located just upstream of tsp-2 was initially associated with tsp-1, as the point of maximal read density was located within the tsp-1 transcript sequence (Figure 7B). We performed qRT-PCR on RNA isolated from ∼150 micro-dissected intestines to quantify tsp-2 expression. Similar to tsp-1, we found that tsp-2 expression is induced in old adults but not induced to the same degree in old adults fed unc-62 RNAi. Bars indicate expression in biological replicate experiments, relative to expression in young adults (normalized to control gene tbb-2). Error bars indicate standard deviation from triplicate technical replicates. (TIF) [file pgen.1003325.s011.tif]
